# Supplementary material for: Gold Nanoparticles Modification with Liquid Crystalline Polybenzylic Dendrons via 1,3-Dipolar Cycloaddition
Source: Nanomaterials (Basel). 2022 Nov 16;12(22):4026. doi: 10.3390/nano12224026 (PMC9699240; doi:10.3390/nano12224026)
Supplement: Supplementary file 1 [file nanomaterials-12-04026-s001.zip › nanomaterials-2029637-supplementary.pdf]

Supplementary Materials

Gold Nanoparticles Modification with Liquid Crystalline Polybenzylic Dendrons via 1,3-Dipolar Cycloaddition

José Antonio Ulloa [1], Joaquín Barberá [2] and José Luis Serrano [2,*]

[1] Departamento de Polímeros, Facultad de Ciencias Químicas, Universidad de Concepción, Casilla 160-C, Calle Edmundo Larenas 129, Concepción 4070371, Chile
[2] Departamento de Química Orgánica, Facultad de Ciencias, Instituto de Nanociencia y Materiales de Aragón (INMA), CSIC-Universidad de Zaragoza, 50009 Zaragoza, Spain
* Correspondence: joseluis@unizar.es; Tel.: +34976761209

CONTENTS

S1. Materials and methods. Abreviations. **page S3**

S2. Experimental procedures. **page S5**

S2.1 Synthesis and characterization of the dendronic structures. **page S5**

S2.1.1. ***Scheme S1***. Synthetic route of the precursors ***1-9***. **page S5**

S2.1.2. ***Scheme S2***. Synthetic route of the precursors ***10-21***. **page S8**

S2.1.3. ***Scheme S3***. Synthetic route of the alkyne focal-point dendrons (Compounds ***22-27***). **page S11**

S2.1.4. Chemical characterization of the dendrons **page S14**

S2.2. Synthesis of 11-azidoundecane-1-thiol (***Scheme S4***). **page S16**

S2.3 Synthesis and characterization of ***AuNP***. **page S17**

S2.3.1 Synthesis of gold nanoparticles and ligand exchange reaction (***Scheme S5***). **page S17**

S2.3.2. Functionalization of ***11-azidoundecane-1-thiol*** gold nanoparticles (***AuDT-AT***) with alkynyl focal-point dendrons by click reaction (***Scheme S6***). **page S18**

S3. Mesogenic and optical characterization of the alkynyl focal-point dendrimers. **page S20**

S3.1 Thermogravimetric analysis of alkynyl focal-point dendrons. **page S20**

S3.2. Optical textures of alkynyl focal-point dendrons.

S3.3. **XRD** studies of alkynyl focal-point dendrons. **page S21**

S3.4 **UV-Vis** studies of alkynyl focal-point dendrons. **page S23**

S4. Structural characterization of the ***AuDT -TA@Ln*** nanoparticles. **page S24**

S4.1. ^{1}**H-NMR** spectra of ***AuDT***, ***AuDT-AT*** and ***AuDT-TA@Ln*** gold nanoparticles. **page S24**

S4.2. **UV-Vis** studies of ***AuDT-TA@Ln***. **page S28**

S4.3. **XPS** studies of ***AuDT-TA@Ln***. **page S29**

S5. Characterization of the gold nanoparticles after the isothermal treatment. **page S30**

S5.1. **TEM** exploratory study of the isothermal treatment of the ***AuDT-TA@L2-3,4*** nanoparticles. **page S30**

S5.2. **TEM** characterization of the gold nanoparticles after a thermal treatment at 150 ^{o}C for 0, 30, 60, 120 and 180 minutes. **page S33**

S5.3. Selected area electron diffraction (**SAED**) studies of ***AuDT-TA@Ln***. **page S34**

S5.4. Ligand percentages in the ***AuDT-TA@Ln*** nanoparticles. **page S37**

S5.5. ***Table S13***: **UV-Vis** data of ***AuDT-TA@Ln***. **page S38**

S1. Materials and methods

All reagents, silica gel were purchased from Sigma-Aldrich®. Anhydrous THF and DCM were purchased from Scharlab and dried using a solvent purification system.

The chemical characterization of all the compounds was carried out by FTIR spectroscopy, ^{1}H- and ^{13}C NMR and MALDI-TOF mass spectroscopy. Size exclusion chromatography (SEC) was performed with Sephadex LH-20 purchased from GE Healthcare Life Science®.

FTIR spectra were obtained using a Thermo NICOLET Avatar 360 FT-IR spectrophotometer and Bruker Vertex 70 MKII Golden Gate Single Reflection ATR System. NMR experiments were carried out on a Bruker AV-400 spectrometer operating at 400 MHz for ^{1}H and 100 MHz for ^{13}C equipped with a QNP probe (Quattro nucleus probe). Chemical shifts are given in ppm relative to TMS and this was used as internal reference. MALDI-TOF MS was performed on an Autoflex Mass Spectometer Bruker Daltonics apparatus using ditranol as matrix.

X-ray photoelectron spectra (XPS) were recorded on a Kratos Axis Supra XPS system with a base pressure of 1×10^{-10} mbar using a monochromated Al Kα X-ray source. XPS survey scans were taken at a pass energy of 225 W (15mA/ 15kV). Data analysis was done with the CASA XPS software package.

Thermogravimetric analysis (TGA) was performed using a Q5000IR apparatus from TA Instruments at a heating rate 10 °C min^{-1} under nitrogen atmosphere. Thermal transitions were determined by differential scanning calorimetry (DSC) using a Q2000 equipment from TA Instruments. Mesogenic behavior was investigated by polarized-light optical microscopy (POM) using an Olympus BH-2 polarizing microscope fitted with a Linkam THMS600 hot stage.

X-ray diffraction (XRD) was performed with an evacuated pinhole camera (Anton-Paar) operating a point-focused Ni-filtered Cu-Kα beam. Powdered samples of the alkyne focal-point dendrons were placed in Lindemann glass capillaries (0.9 mm diameter). The patterns were collected on flat photographic film perpendicular to the X-ray beam. An Anton-Paar high-temperature attachment was used when necessary.

Transmission electronic microscopy (TEM) images and selected area electron diffraction (SAED) patterns were obtained by FEI Tecnai TF20 in a 200kV FEG high resolution Transmission Electron. Data analysis was done with Image-J software package.

UV-Vis absorption spectroscopy was performed using an ATI-UNICAM UV4-200 spectrophotometer with quartz cuvette 10 mm pathlength.

Abreviations.

AT: 11-azidoundecane-1-thiol
ATR-FTIR: Attenuated total reflectance - Fourier-transform infrared spectroscopy
AuDT: Dodecanethiol-coated gold nanoparticles
AuDT-AT: AuDT nanoparticles after ligand exchange with 11-azidoundecane-1-thiol
AuNP: Gold nanoparticle
DCM: Dichloromethane
DMF: N,N-Dimethylformamide
DSC: Differential scanning calorimetry
DT: Dodecane-1-thiol
FTIR: Fourier-transform infrared spectroscopy
HRXPS: High –resolution X-ray photoelectron spectroscopy
MALDI-TOF: Matrix-assisted laser desorption/ionization – Time of flight
NMR: Nuclear magnetic resonance
NP: Nanoparticle
POM: Polarized optical microscopy
SAED: Selected-area electron diffraction
SEC: Size exclusion chromatography
SPR: Surface plasmon resonance
STEM: Scanning transmission electron microcopy
TBTA: Tris(benzyltriazolyl)methyl amine
TEM: Transmission electron microcopy
TGA: Thermogravimetric analysis
THF: Tetrahydrofuran
UV-vis: Ultraviolet-visible
XPS: X-ray photoelectron spectroscopy
XRD: X-ray diffraction

S2. Experimental Procedures

S2.1 Synthesis and characterization of the dendronic structures.

S2.1.1. Scheme S1. Synthetic route of the precursors 1-9.

In the ***Scheme S1*** are represented the first three steps of the synthetic route of the dendronic ligands (Compounds 1-9).

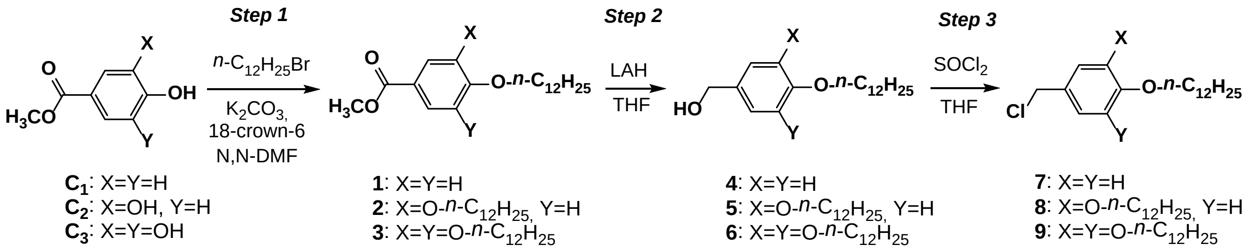

***Scheme S1**: Synthetic route of precursors 1-9*

S2.1.1.1. ***Scheme S1-step 1.*** **(Compounds 1-3).**

General procedure of Williamson reaction.

A mixture of the corresponding phenol or polyphenol ester (1 eq.), anhydrous K_2CO_3 (1.3 eq. per OH) and a teaspoon of 18-crown-6 in *N,N*-dimethylformamide (DMF) was stirred and heated. The halide (1.1 eq. per OH) was added dropwise to the resulting solution and the mixture was stirred and heated at 110 °C for 36 - 48 h. The mixture was allowed to cool to room temperature and then it was poured into water. The crude product was extracted with ethyl acetate (AcOEt). The organic phase was washed with brine, dried over $MgSO_4$, filtered and the solvent was evaporated by a rotatory evaporator system.

Methyl 4-*n*-dodecyloxybenzoate (1): White solid. Yield: 86 %. **^{1}H-NMR** (400 MHz, $CDCl_3$): δ = 0.81 (t, *J* = 6.9 Hz, 3H), 1.19 (m, 16H), 1.38 (m, 2H), 1.72 (m, 2H), 3.81 (s, 3H), 3.93 (t, *J* = 6.6 Hz, 2H), 6.83 (m, 2H), 7.90 (m, 2H) ppm. **^{13}C-NMR** (100 MHz, $CDCl_3$): δ = 14.11, 22.69, 25.98, 29.35, 29.36, 29.56, 29.59, 29.63, 29.66, 68.21, 114.07, 122.21, 131.56, 162.94, 166.94 ppm. **IR**: ν = 2924, 2854, 1721, 1606, 1579 cm^{-1}. **MS** (ESI^+) m/z: 321.2 $[M+H]^+$.

Ethyl 3,4-bis(*n*-dodecyloxy)benzoate (2): White solid. Yield: 83 %. **^{1}H-NMR** (400 MHz, $CDCl_3$): δ = 0.90 (t, *J* = 6.9 Hz, 6H), 1.36 (m, 36H), 1.49 (m, 4H), 1.85 (m, 4H), 3.96 (t, *J* = 6.6 Hz, 4H) 4.36 (c, *J* = 7.1 Hz, 2H), 6.88 (d, *J* = 8.0 Hz, 1H), 7.56 (d, *J* = 2.0 Hz, 1H), 7.66 (dd, J_1 = 8.4 Hz, J_2 = 2.0 Hz,

1H) ppm. **^{13}C-NMR** (100 MHz, $CDCl_3$): δ = 14.13, 14.42, 22.71, 29.38, 29.40, 29.43, 29.62, 29.64, 29.67, 29.71, 29.72, 31.94, 60.70, 69.01, 69.29, 111.90, 114.29, 122.77, 123.45, 148.49, 153.13, 166.55 ppm. **IR**: ν = 2924, 2854, 1717, 1599, 1510 cm^{-1}. **MS** ($MALDI^+$) m/z (%): 541.4 (95.9%) $[M+Na]^+$.

Methyl 3,4,5-tri(*n*-dodecyloxy)benzoate (3): White solid. Yield: 79 %. **^{1}H-NMR** (400 MHz, $CDCl_3$): δ = 0.89 (m, 9H), 1.28 (m, 42H), 1.75 (m, 6H), 3.89 (s, 3H), 4.02 (m, 6H), 7.26 (s, 2H) ppm. **RMN-^{13}C** (100 MHz, $CDCl_3$): δ 14.1, 22.7, 26.0, 29.2, 29.3, 29.5, 30.3, 31.9, 69.1, 73.4, 107.9, 124.6, 152.7, 167.0 ppm. **IR**: ν = 2921, 2852, 1718, 1588 cm^{-1}. **MS** ($MALDI^+$) m/z (%): 711.15 (100%) $[M+Na]^+$.

S2.1.1.2. ***Scheme S1-step 2. (Compounds 4-6).***

General procedure for the synthesis of benzyl alcohols.

The benzyl alcohols were synthesized by the reduction of the corresponding ester with $LiAlH_4$ (LAH). In a flask a suspension of $LiAlH_4$ (1 eq.) in dry THF was stirred under argon atmosphere. The ester (1 eq.) dissolved in dry THF was added dropwise and the mixture was stirred for 2 h at room temperature. The reaction mixture was quenched by addition of $Na_2SO_4 \cdot 10H_2O$. The salt was filtered off upon celite, washed with THF and the solvent was removed by rotary evaporation. The crude was dissolved in dichloromethane (DCM) and washed with brine. The organic phase was dried over $MgSO_4$, filtered and the solvent was removed by rotary evaporation.

4-*n*-dodecyloxybenzyl alcohol (4): White solid. Yield: 94 %. **^{1}H-NMR** (400 MHz, $CDCl_3$): δ = 0.88 (t, *J* = 6.5 Hz, 3H), 1.26 (m, 18H), 1.77 (m, 2H), 3.95 (t, *J* = 6.6 Hz, 2H), 4.61 (s, 2H), 6.88 (d, *J* = 8.8 Hz, 2H), 7.28 (d, *J* = 8.8 Hz, 2H) ppm. **^{13}C-NMR** (100 MHz, $CDCl_3$): δ = 14.13, 22.70, 26.05, 29.27, 29.37, 29.41, 29.59, 29.61, 29.64, 29.66, 31.93, 65.15, 68.10, 114.59, 128.65 ppm. **IR**: ν = 3150, 2923, 2852 1610, 1580, 1280 cm^{-1}. **MS** (ESI^+) m/z: 275.1 $[M\text{-}OH]^+$.

3,4-di(*n*-dodecyloxy)benzyl alcohol (5): White solid. Yield: 91 %. **^{1}H-NMR** (400 MHz, $CDCl_3$): δ = 0.81 (t, *J* = 6.9 Hz, 6H), 1.23 (m, 32H), 1.38 (m, 4H), 1.73 (m, 4H), 3.92 (q, J_1 = 8.0 Hz, J_2 = 4.0 Hz, 4H), 4.52 (s, 2H), 6.78 (d, J_1 = 0.9 Hz, 2H), 6.85 (s, 1H) ppm. **^{13}C-NMR** (100 MHz, $CDCl_3$): δ = 14.12, 22.70, 25.61, 26.06, 29.33, 29.38, 29.45, 29.65, 29.68, 29.72, 31.94, 65.39, 67.97, 69.26, 69.46, 113.03, 113.92, 119.60, 129.44, 133.75, 148.76, 149.38 ppm. **IR**: ν = 3248, 2954, 2919, 2849, 1592, 1238, 1020 cm^{-1}. **MS** ($MALDI^+$) m/z (%): 499.4 (100%) $[M+Na]^+$.

3,4,5-tri(*n*-dodecyloxy)benzyl alcohol (6). White solid. Yield 87 %. **^{1}H-NMR** (400 MHz, $CDCl_3$): δ = 0.90 (t, *J* = 6.9 Hz, 9H), 1.30 (m, 48H), 1.47 (m, 6H), 1.78 (m, 6H), 3.97 (m, 6H), 4.61 (d, *J* = 4.7 Hz, 2H), 6.57 (s, 2H) ppm. **^{13}C-NMR** (100 MHz, $CDCl_3$): δ = 14.13, 22.71, 26.12, 29.38, 29.43, 29.67, 29.72, 29.76, 29.77, 30.34, 31.96, 65.70, 69.11, 73.44, 105.35, 136.03, 137.61, 153.30 ppm. **IR**: ν = 3306, 2922, 2852, 1591 cm^{-1}. **MS** ($MALDI^+$) m/z (%): 683.6 (97.5%) $[M+Na]^+$.

S2.1.1.3. ***Scheme S1-step 3. (Compounds 7-9).***

General procedure for the synthesis of benzyl chlorides.

In a dry flask under argon atmosphere the benzyl alcohol (1 eq.) was dissolved in dry DCM and the solution was cooled in a water/ice bath for 15 minutes. A solution of $SOCl_2$ (1.2 eq.), dry triethylamine (TEA, 1.5 eq) and a catalytic amount of DMF was added dropwise. After the addition was completed, the mixture was stirred overnight at 50 °C. The reaction was quenched by addition of water and the organic solution was washed three times with water. The organic phase was dried over $MgSO_4$, filtered and the solvent was removed by rotary evaporation.

4-*n*-dodecyloxybenzyl chloride (7): Yellow solid. Yield: quantitative. **^{1}H-NMR** (400 MHz, $CDCl_3$): δ = 0.88 (t, *J* = 6.7 Hz, 3H), 1.27 (m, 18H), 1.74 (m, 2H), 3.95 (t, *J* = 6.6 Hz, 2H), 4.57 (s, 2H), 6.87 (d, *J* = 8.9 Hz, 2H), 7.30 (d, *J* = 8.7 Hz, 2H) ppm. **^{13}C-NMR** (100 MHz, $CDCl_3$): δ = 14.11, 22.69, 26.03, 29.23, 29.35, 29.57, 2960, 29.64, 29.66, 31.92, 46.37, 68.11, 114.71, 130.01, 159.31 ppm. **IR**: ν = 2924, 2854 1610, 1510, 1250 cm^{-1}. **MS** (ESI^+) m/z: 311.1 $[M+H]^+$.

3,4-di(*n*-dodecyloxy)benzyl chloride (8): Yellow solid. Yield: 96 %. **^{1}H-NMR** (400 MHz, $CDCl_3$): δ = 0.91 (t, *J* = 6.8 Hz, 6H), 1.33 (m, 32H), 1.49 (m, 4H), (4.57 (m, 4H), 4.02 (m, 6H), 4.57 (s, 2H), 6.84 (m, 1H), 6.90 (m 1H), 6.93 (t, *J* = 2.3 Hz, 1H) ppm. **^{13}C-NMR** (100 MHz, $CDCl_3$): δ = 14.12, 22.71, 26.04, 26.05, 29.26, 29.29, 29.38, 29.43, 29.44. 29.65, 29.68, 29.72, 31.94, 46.76, 69.30, 113.55, 114.29, 121.27, 130.02, 149.30, 149.44 ppm. **IR**: ν = 2924, 2853, 1588, 1512, 1264, 1024 cm^{-1}. **MS** ($MALDI^+$) m/z (%): 517.4 (15.7%) $[M+Na]^+$.

3,4,5-tri(*n*-dodecyloxy)benzyl chloride (9): White solid. Yield 96 %. **^{1}H-NMR** (400 MHz, $CDCl_3$): δ = 0.81 (t, *J* = 6.9 Hz, 9H), 1.21 (m, 48H), 1.39 (m, 6H), 1.69 (m, 6H), 3.88 (m, 6H), 4.44 (s, 2H), 6.49 (s, 2H) ppm. **^{13}C-NMR** (100 MHz, $CDCl_3$): δ = 14.13, 22.71, 29.39, 29.42, 29.66, 29.68, 29.72, 31.95, 47.00, 69.14, 73.45, 107.08, 132.31, 138.32, 153.22 ppm. **IR**: ν =2923, 2853, 1590, 1235 cm^{-1}. **MS** ($MALDI^+$) m/z (%): 701.6 (100 %) $[M+Na]^+$.

S2.1.2. Scheme S2. Synthetic route of the precursors 10-21.

In the ***Scheme S2*** are summarized the synthetic route of the dendronic precursors 10-21.

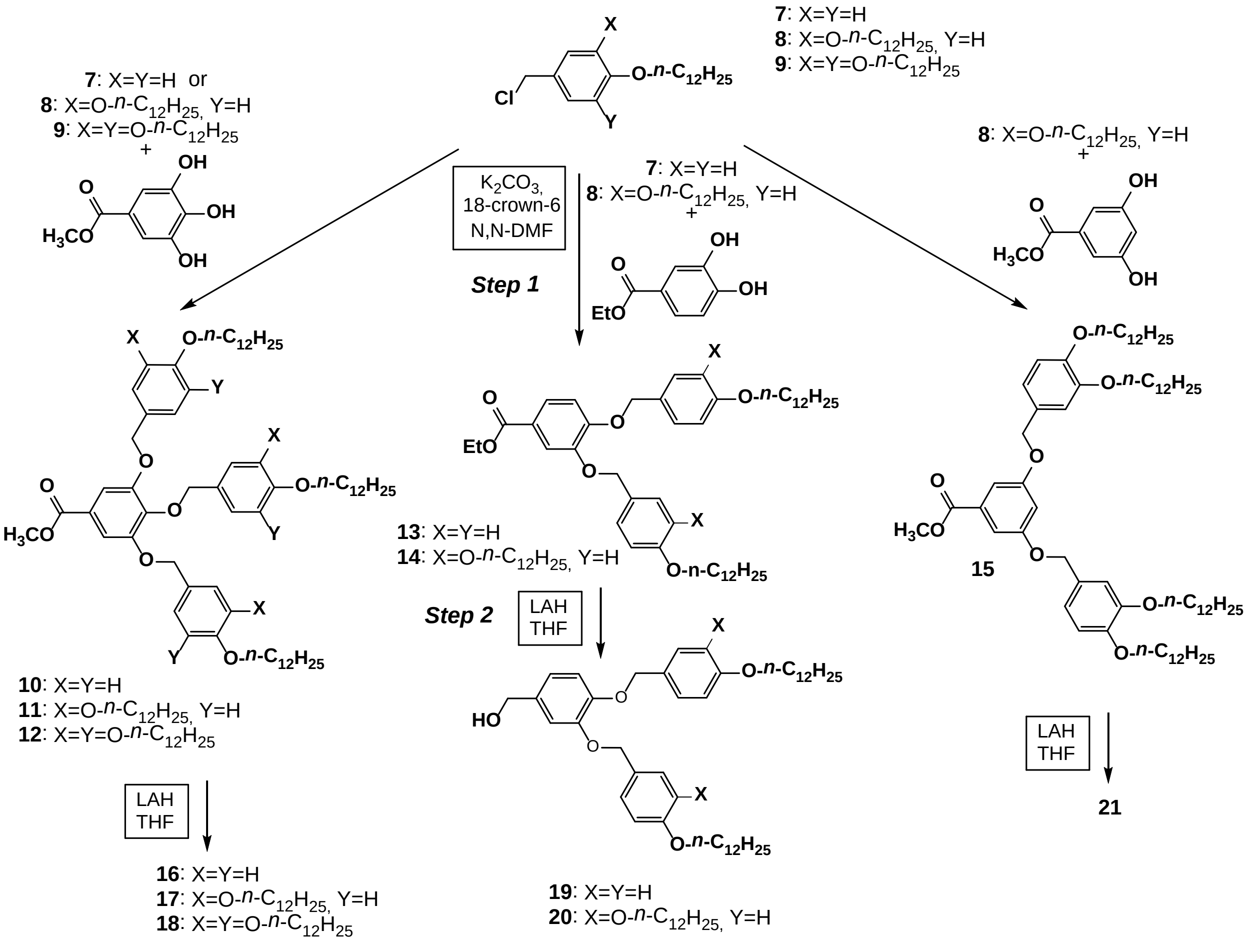

***Scheme S2**: Synthetic route of dendronic precursors 10-21*

S2.1.2.1. ***Scheme S2-step 1. (Compounds 10-15).***

General procedure for the synthesis of the dendritic ester precursors.

A mixture of the corresponding phenol or polyphenol ester (1 eq.), anhydrous K2CO3 (1.3 eq. per OH) and a teaspoon of 18-crown-6 in N,N-dimethylformamide (N,N-DMF) was stirred and heated. 1-bromododecane (1.1 eq. per OH) was added dropwise to the resulting solution and the mixture was stirred and heated at 110 ºC for 36-48 h. The mixture was allowed to cool to room temperature and then it was poured into water. The crude product was extracted with ethyl acetate (AcOEt). The organic phase was washed with brine and dried over $MgSO_4$, filtered and the solvent was evaporated using a vacuum rotary evaporator.

Methyl 3,4,5-tris(4-*n*-dodecyloxybenzoxy)benzoate (10): White solid. Yield: 52 %. **^{1}H-NMR** (400 MHz, $CDCl_3$): δ = 0.81 (t, *J* = 6.9 Hz, 9H), 1.23 (m, 48H), 1.38 (m, 6H), 1.70 (m, 6H), 3.81 (s, 3H), 3.83 (t, *J* = 8.0 Hz, 2H), 3.88 (t, *J* = 4.0 Hz, 4H), 4.93 (s, 2H), 4.96 (s, 4H), 6.67 (m, *2*H), 6.82 (m, 4H), 7.17 (m, 2H), 7.26 (m, 2H), 7.29 (s, 2H) ppm. **^{13}C-NMR** (100 MHz, $CDCl_3$): δ = 14.13, 22.70, 26.09, 29.32, 2934, 29.37, 29.45, 29.47, 29.61, 29.63, 29.66, 29.69, 31.94, 65.49, 68.00, 68.09, 71.19, 74.81, 106.96, 114.12, 114.45, 129.04, 129.13, 129.93, 130.27, 136.40, 153.09, 158.98 ppm. **IR**: ν = 2919, 2850, 1717, 1594, 1515, 1246 cm^{-1}. **MS** ($MALDI^+$) m/z (%): 1029.9 (100%) $[M+Na]^+$.

Methyl 3,4,5-tris(3,4-bis(*n*-dodecyloxy)benzoxy)benzoate (11): White solid. Yield 84 %. **^{1}H-NMR** (400 MHz, $CDCl_3$): δ = 0.90 (t, *J* = 6.0 Hz, 18H), 1.28 (m, 96H), 1.47 (m, 12H), 1.78 (m, 12H), 3.90 (s, 3H), 3.96 (m, 8H), 4.01 (t, *J* = 6.7 Hz, 4H), 5.04 (s, 2H), 5.06 (s, 4H), 6.74 (d, *J* = 8.2 Hz, 1H), 6.86 (m, 2H), 6.94 (d, *J* = 1.8 Hz, 1H), 6.96 (dd, J_1 = 5.5 Hz, J_2 = 1.9, 2H), 7.01 (d, *J* = 1.9 Hz, 2H), 7.40 (s, 2H) ppm. **^{13}C-NMR** (100 MHz, $CDCl_3$): δ = 14.13, 22.71, 26.10, 29.36, 29.38, 29.40, 29.43, 29.51, 29.54, 29.59, 29.70, 29.74, 29.75, 29.80, 31.95, 52.18, 68.94, 69.23, 69.33, 71.39, 109.41, 113.40, 113.62, 114.12, 120.28, 121.08, 125.08, 129.30, 130.15, 142.51, 149.00, 149.25, 152.65, 166.67 ppm. **IR**: ν = 2922, 2853, 1717, 1590, 1516, 1228, 1015 cm^{-1}. **MS** ($MALDI^+$) m/z (%): 663.4 (100 %) [$C_{39}H_{60}O_7$, Na^+], 1583.4 (36.8 %) [$C_{101}H_{170}O_{11}$, Na^+].

Methyl 3,4,5-tris(3,4,5-(*n*-dodecyloxy)benzoxy)benzoate (12): White solid. Yield 78 %. **^{1}H-NMR** (400 MHz, $CDCl_3$): δ = 0.80 (t, *J* = 6.7 Hz, 27), 1.18 (m, 144H), 1.35 (m, 18H), 1.64 (m, 18H), 3.73 (s, 3H), 3.81 (t, *J* = 6.5 Hz, 18H), 4.95 (s, 4H), 4.96 (s, 2H), 6.53 (s, 2H), 6.56 (s, 4H), 7.31 (s, 2H) ppm. **^{13}C-NMR** (100 MHz, $CDCl_3$): δ = 14.11, 22.71, 26.18, 29-.41, 29.43, 29.52, 29.71, 29.73, 29.76, 29.80, 31.97, 67.97, 68.91, 69.09, 71.68, 73.32, 73.40, 75.15, 105.75, 106.28, 109.64, 113.88, 125.25, 128.50, 129.43, 131.67, 132.40, 137.89, 152.58, 153.03, 153.29, 166.53 ppm. **IR**: ν = 2921, 2851, 1721, 1590 cm^{-1}. **MS** ($MALDI^+$) m/z (%): 2135.9 (100 %) $[M+Na]^+$.

Ethyl 3,4-bis(4-(*n*-dodecyloxy)benzoxy)benzoate (13): White solid. Yield 57 %. **^{1}H-NMR** (400 MHz, $CDCl_3$): δ = 0.81 (t, *J* = 6.9 Hz, 6H), 1.25 (m, 35H), 1.37 (m, 4H), 1.70 (m, 4H), 3.87 (t, *J* = 8.0 Hz, 4H), 4.25 (m, 2H), 5.02 (s, 2H), 5.04 (s, 2H), 6.80 (d, *J* = 8.0 Hz, 4H), 6.85 (d, *J* = 12 Hz, 1H), 7.25 (m, 4H), 7.53 (d, *J* = 2.0 Hz, 1H), 7.56 (dd, J_1 = 5.7 Hz, J_2 = 2.0 Hz, 2H) ppm. **^{13}C-NMR** (100 MHz, $CDCl_3$): δ = ppm. **IR**: ν = 2920, 2852, 1707, 1599, 1515, 1247, 1029 cm^{-1}. **MS** ($MALDI^+$) m/z (%): 753.6 (90.2%) $[M+Na]^+$.

Ethyl 3,4-bis(3,4-bis(dodecyloxy)benzoxy)benzoate (14): White solid. Yield 95 %. **^{1}H-NMR** (400 MHz, $CDCl_3$): δ = 0.90 (t, *J* = 6.8 Hz, 12H), 1.35 (m, 67), 1.47 (m, 8H), 1.78 (m, 8H), 3.95 (m, 4H), 4.00 (m, 4H), 4.35 (q, *J* = 7.1 Hz, 2H), 5.11 (s, 2H), 5.13 (s, 2H), 6.86 (dd, J_1 = 8.2 Hz, J_2 = 2.1, 2H), 6.94 (m, 2H), 6.99 (m, 2H), 7.03 (d, *J* = 1.9 Hz, 1H), 7.64 (d, *J* = 2.0 Hz, 1H), 7.67 (dd, J_1 = 4.8 Hz, J_2

= 1.9, 1H) ppm. **^{13}C-NMR** (100 MHz, $CDCl_3$): δ = 14.13, 14.39, 22.71, 26.08, 29.35, 29.39, 29.40, 29.49, 29.51, 29.69, 29.70, 29.73, 29.75, 31.95, 60.75, 69.23, 69.34, 70.95, 71.32, 113.11, 113.38, 113.66, 115.71, 119.95, 120.23, 123.37, 123.88, 129.14, 129.47, 148.37, 149.00, 149.26, 149.31, 152.95, 166.32 ppm. **IR**: ν = 2923, 2853, 1714, 1599, 1516, 1267, 1027 cm^{-1}. **MS** ($MALDI^+$) m/z (%): 1122.0 (100 %) [$C_{71}H_{118}O_8$, Na^+].

3,4,5-tris(4-*n*-dodecyloxybenzoxy)benzyl alcohol (15): White solid. Yield: 91 %. **^{1}H-NMR** (400 MHz, $CDCl_3$): δ = 0.81 (t, *J* = 6.9 Hz, 9H), 1.23 (m, 48H), 1.37 (m, 6H), 1.69 (m, 6H), 3.87 (m, 6H), 3.88 (t, *J* = 4.0 Hz, 4H), 4.86 (s, 2H), 4.92 (s, 4H), 6.57 (m, *2*H), 6.69 (m, 2H), 6.81 (m, 4H), 7.20 (m, 2H), 7.24 (m, 4H) ppm. **^{13}C-NMR** (100 MHz, $CDCl_3$): δ = 14.14, 22.71, 26.09, 29.32, 29.37, 29.45, 29.62, 29.63, 29.66, 29.69, 31.94, 65.21, 68.06, 71.18, 71.38, 114.39, 114.35, 114.46, 115.56, 120.14, 129.02, 129.08, 129.11, 134.41, 148.63, 149.34 158.94 ppm. **IR**: ν = 3306, 2918, 2851, 1515, 1247 cm^{-1}. **MS** ($MALDI^+$) m/z (%): 1001.7 (100%) $[M+Na]^+$.

S2.1.2.2. Scheme S2-step 2. (Compounds 16-21).

General procedure for the synthesis of the benzyl alcohol dendronic precursors.

In a flask a suspension of $LiAlH_4$ (1 eq.) in dry THF was stirred under argon atmosphere. The ester (1 eq.) dissolved in dry THF was added dropwise and the mixture was stirred for 2 h at room temperature. The reaction was quenched by addition of $Na_2SO_4 \cdot 10H_2O$. The salt was filtered off upon celite, washed with THF and the solvent was removed by rotary evaporation. The crude was dissolved in DCM and washed with brine. The organic phase was dried over $MgSO_4$, filtered and the solvent was removed by rotary evaporation.

3,4,5-tris(3,4-bis(*n*-dodecyloxybenzoxy)benzyl alcohol (16): White/yellow solid. Yield: 82 %. **^{1}H-NMR** (400 MHz, $CDCl_3$): δ = 0.90 (t, *J* = 6.0 Hz, 18H), 1.28 (m, 96H), 1.46 (m, 12H), 1.80 (m, 12H), 3.94 hg(m,6H), 3.99 (t, *J* = 6.4 Hz, 6H), 4.59 (d, *J* = 5.3 Hz, 2H), 4.97 (s, 2H), 5.04 (s, 4H), 6.68 (m, 2H), 6.76 (d, *J* = 8.2 Hz, 1H), 4.01 (t, *J* = 6.7 Hz, 4H), 5.04 (s, 2H), 5.06 (s, 4H), 6.74 (d, *J* = 8.2 Hz, 1H), 6.86 (m, 2H), 6.94 (d, *J* = 1.8 Hz, 1H), 6.85 (d, *J* = 8.2 Hz, 2H), 6.89 (dd, J_1 = 8.2 Hz, J_2 = 1.9 Hz, 2H), 7.01 (m, 3H) ppm. **^{13}C-NMR** (100 MHz, $CDCl_3$): δ = 14.11, 22.70, 26.10, 29.39, 29.50, 29.51, 29.53, 29.59, 29.69, 29.72, 29.74, 29.78, 31.94, 65.45, 68.94, 69.25, 69.39, 71.40, 75.01, 106.87, 133.36, 113.52, 113.71, 114.22, 120.11, 121.05, 129.81, 130.65, 136.45, 137.95, 148.91, 149.28, 153.08 ppm. **IR**: ν = 3301, 2922, 2852, 1591, 1517, 1234, 1102 cm^{-1}. **MS** ($MALDI^+$) m/z (%): 635.4 (100 %) [$C_{38}H_{60}O_6$, Na^+], 1555.2 (9.37 %) [$C_{100}H_{170}O_{10}$, Na^+].

3,4,5-tris(3,4,5-tris(*n*-dodecyloxy)benzoxy)benzyl alcohol (17): White/yellow solid. Yield: 85 %. **^{1}H-NMR** (400 MHz, $CDCl_3$): δ = 0.90 (t, *J* = 6.8 Hz, 27H), 1.28 (m, 144H), 1.45 (m, 18H), 1.74 (m, 18H), 3.92 (m, 18H), 4.99 (s, 2H), 5.03 (s, 4H), 6.64 (s, 4H), 6.65 (s, 2H), 6.69 (s, 2H) ppm. **^{13}C-**

NMR (100 MHz, $CDCl_3$): δ = 14.49, 22.31, 26.80, 30.10, 30.13, 30.22, 30.32, 30.38, 30.41, 31.02, 31.06, 32.56, 65.64, 69.37, 69.54, 72.04, 73.82, 73.89, 75.65, 106.09, 106.42, 106.97, 132.77, 133.62, 137.75, 138.06, 138.10, 138.19, 153.50, 153.59, 153.84 ppm. **IR**: ν = 374, 2924, 2853, 1589, 1234, 1115 cm^{-1}. **MS** ($MALDI^+$) m/z (%): 2107.5 (62.2%) $[M+Na]^+$.

3,4-bis(4-*n*-dodecyloxybenzoxy)benzyl alcohol (18): White solid. Yield: 79 %. **^{1}H-NMR** (400 MHz, $CDCl_3$): δ = 0.81 (t, *J* = 6.9 Hz, 6H), 1.25 (m, 35H), 1.38 (m, 4H), 1.70 (m, 4H), 3.87 (t, *J* = 6.6 Hz, 4H), 4.49 (m, 2H), 4.98 (s, 2H), 4.99 (s, 2H), 6.79 (m, 6H), 6.91 (d, *J* = 1.9 Hz, 1H), 7.25 (m, 4H) ppm. **^{13}C-NMR** (100 MHz, $CDCl_3$): δ = 14.14, 22.71, 26.08, 29.31, 29.37, 29.45, 29.61, 29.63, 29.66, 29.69, 31.94, 65.24, 68.05, 71.17, 71.36, 114.37, 114.44, 114.46, 115.53, 120.14, 129.01, 129.08, 129.16, 134.37, 148.63, 149.33, 158.93 ppm. **IR**: ν = 3273, 2922, 2853, 1518, 1248 cm^{-1}. **MS** ($MALDI^+$) m/z (%): 711.5 (87.4%) $[M+Na]^+$.

3,4-bis(3,4-bis(*n*-dodecyloxy)benzoxy)benzyl alcohol (19): White solid. Yield: 66 %. **^{1}H-NMR** (400 MHz, $CDCl_3$): δ = 0.90 (t, *J* = 6.8 Hz, 12H), 1.30 (m, 64H), 1.47 (m, 8H), 1.81 (m, 8H), 3.96 (m, 8H), 4.59 (bs, 1H), 5.08 (m, 4H), 6.85 (m, 3H), 6.94 (m, 3H), 7.01 (m, 3H) ppm. **^{13}C-NMR** (100 MHz, $CDCl_3$): δ = 14.12, 22.70, 26.08, 29.35, 29.36, 29.38, 29.39, 29.67, 29.69, 29.72, 29.74, 31.94, 65.26, 69.21, 69.37, 110.00, 113.31, 113.36, 113.42, 113.71, 114.36, 120.06, 120.11, 120.18, 123.38, 129.86, 129.93, 139.39, 148.38, 148.64, 148.87, 148.88, 149.27 ppm. **IR**: ν = 3305, 2920, 2851, 1591, 1519, 1265, cm^{-1}. **MS** ($MALDI^+$) m/z (%): 1080.0 (100 %) [$C_{69}H_{116}O_7$, Na^+].

S2.1.3. Scheme S3. Synthetic route of the alkyne focal-point dendrons (Compounds 22-27).

S2.1.3.1 In the ***Scheme S3*** are represented the synthetic route of the alkyne focal point dendrons by means of the **Steglish esterification** (Compounds 22-27).

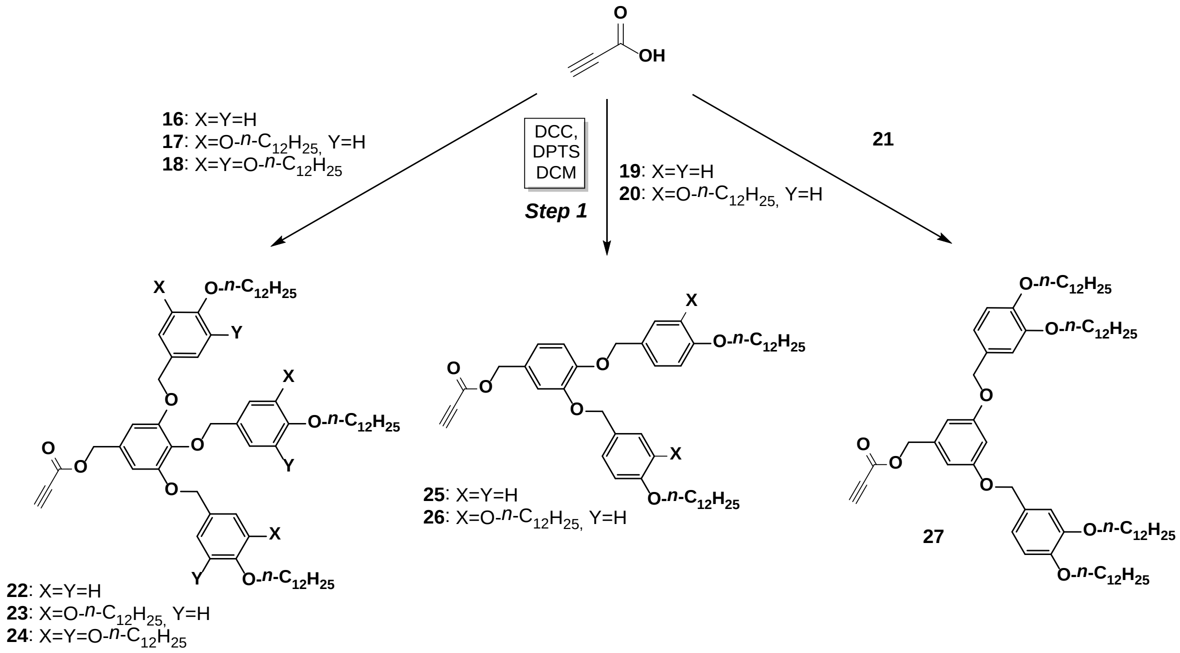

***Scheme S3**: Synthetic route of alkyne focal point dendrons*

S2.1.3.2 General procedure.

In a dry flask under argon atmosphere propiolic acid (1 eq.) was added dropwise upon a mixture of the corresponding benzyl alcohol (1 eq.) and 4-(dimethylamino)pyridinium *p*-toluenesulfonate (DPTS, 0.8 eq.) dissolved in dry DCM. The mixture was cooled in a water/ice bath for 15 minutes; then a solution of *N,N*-dicyclohexylcarbodiimide (DCC, 1.1 eq.) was added dropwise at 0 ºC. The mixture was stirred at room temperature for 24 - 48 h under an argon atmosphere. The white precipitate was filtered off and the solvent was evaporated. Finally, the crude product was purified by column chromatography on silica gel.

3,4,5-tris[4-(dodecyloxy)benzoxi]benzyl propiolate (22): White solid. Yield: 50 %. **^{1}H-NMR** (300 MHz, $CDCl_3$): δ = 0.88 (t, *J* = 6.8 Hz, 9H), 1.27 (m, 48H), 1.43 (m, 6H), 1.76 (m, 6H), 2.98 (s, 1H), 3.91 (t, *J* = 6.6 Hz, 2H), 3.96 (t, *J* = 6.6 Hz, 4H), 4.88 (s, 2H), 4.99 (s, 4H), 5.10 (s, 2H), 6.68 (S, *2*H), 6.74 (m, 2H), 6.89 (dd, J_1 = 6.8 Hz, J_2 = 1.8 Hz, 4H), 7.23 (d, *J* = 8.5 Hz, 2H), 7.24 (m, 4H), 7.33 (dd, J_1 = 8.4 Hz, J_2 = 1.6 Hz, 4H), ppm. **^{13}C-NMR** (100 MHz, $CDCl_3$): δ = 13.87, 2.69, 26.04, 29.30, 29.32, 29.35, 29.43, 29.45, 29.62, 29.64, 29.67, 31.93, 67.99, 68.09, 70.94, 74.44, 74.65, 74.87, 107.92, 113.96, 114.39, 128.74, 129.35, 129.98, 130.12, 138.47, 152.34, 153.00, 159.01, 159.16 ppm. **IR**: ν = 3264, 2922, 2852, 2118, 1713, 1515, 1246, 1107 cm^{-1}. **MS** ($MALDI^+$) m/z (%): 1053.7 (39.62 %) [$C_{67}H_{98}O_8$, Na^+].

3,4,5-tris[3,4-bis(dodecyloxy)benzoxi]benzyl propiolate (23): White solid. Yield: 55 %. **^{1}H-NMR** (400 MHz, CD_2Cl_2): δ = 0.90 (t, *J* = 5.6 Hz, 18H), 1.27 (m, 96H), 1.46 (m, 12H), 1.77 (m, 12H), 2.99

(s, 1H), 3.90 (m, 8H), 3.97 (t, *J* = 6.8 Hz, 4H), 4.59 (d, *J* = 5.3 Hz, 2H), 4.91 (s, 2H), 5.00 (s, 4H), 5.11 (s, 2H)6.71 (s, 2H), 6.73 (d, *J* = 8.4 Hz, 1H), 6.82 (d, *J* = 1.6 Hz, 1H), 6.85 (s, 1H), 6.87 (s, 1H), 6.92 (m, 2H), 6.94 (d, *J* = 1.89 Hz, 1H), 6.99 (d, *J* = 1.84 Hz, 2H) ppm. **^{13}C-NMR** (100 MHz, $CDCl_3$): δ = 13.87, 22.69, 26.08, 26.17, 29.37, 29.38, 29.40, 29.48, 29.49, 29.66, 29.67, 29.71, 29.73, 29.77, 31.93, 68.05, 69.13, 69.24, 71.22, 74.39, 74.81, 74.86, 108.10, 113.18, 113.44, 133.50, 114.00, 120.29, 120.85, 129.44, 130.05, 130.46, 138.45, 148.87, 149.03, 149.25, 152.31, 153.00 ppm. **IR**: ν = 3259, 2922, 2853, 1713, 1592, 1517, 1231, 10112 cm^{-1}. **MS** ($MALDI^+$) m/z (%): 1607.4 (5.76%) [$M+Na^+$].

3,4,5-tris[3,4,5-tris(dodecyloxy)benzoxi]benzyl propiolate (24): White solid. Yield: 56 %. **^{1}H-NMR** (400 MHz, CD_2Cl_2): δ = 0.86 (t, *J* = 6.6 Hz, 27H), 1.26 (m, 161H), 1.38 (m, 18H), 1.69 (m, 18H), 2.97 (S, 1H), 3.73 (t, *J* = 6.3 Hz, 4H), 3.86 (m, 14H), 4.93 (s, 2H), 4.99 (s, 4H), 5.09 (s, 2H), 6.58 (s, 2H), 6,61 (s, 4H), 6.69 (s, 2H) ppm. **^{13}C-NMR** (100 MHz, $CDCl_3$): δ = 14.48, 23.31, 26.79, 26.86, 30.00, 30.03, 30.09, 30.12, 30.22, 30.31, 30.36, 30.37, 30.40, 31.02, 32.56, 68.58, 69.37, 69.54, 72.18, 73.82, 73.88, 74.99, 75.52, 75.68, 106.12, 106.47, 109.03, 130.92, 132.52, 133.43, 138.11, 138.25, 139.23, 152.92, 153.58, 153.60, 153.86 ppm. **IR**: ν = 2921, 2851, 2120, 1718, 1591, 1505, 1438, 1231, 1119 cm^{-1}. **MS** ($MALDI^+$) m/z (%): 2159.2 (52.0 %) [$C_{139}H_{242}O_{14}$, Na^+].

3,4-bis[4-(dodecyloxy)benzoxi]benzyl propiolate (25): White solid. Yield: 56 %. **^{1}H-NMR** (400 MHz, $CDCl_3$): δ = 0.93 (t, *J* = 6.7 Hz, 6H), 1.32 (m, 32H), 1.48 (m, 4H), 1.81 (m, 4H), 3.00 (s, 1H), 3.99 (t, *J* = 6.6 Hz, 4H), 5.06 (s, 2H), 5.07 (s, 2H), 5.14 (s, 2H), 6.94 (m, 6H), 7.04 (d, *J* = 1.3 Hz, 1H), 7.37 (m, 4H) ppm. **^{13}C-NMR** (100 MHz, $CDCl_3$): δ = 14.12, 22.69, 26.07, 29.30, 29.35, 29.43, 29.60, 29.61, 29.64, 29.67, 31.92, 67.95, 68.05, 71.15, 71.28, 74.68, 74.90, 77.22, 114.48, 115.09, 116.02, 122.33, 127.59, 128.89, 128.92, 128.95, 129.14, 149.13, 149.57, 152.60, 158.94, 158.96 ppm. **IR**: ν = 3267, 2923, 2853, 2119, 1715, 1517, 1246, 1137 cm^{-1}. **MS** ($MALDI^+$) m/z (%): 763.9 (72.6 %) [$C_{48}H_{68}O_6$, Na^+].

3,4-bis[3,4-bis(dodecyloxy)benzoxi]benzyl propiolate (26): White solid. Yield: 45 %. **^{1}H-NMR** (400 MHz, $CDCl_3$): δ = 0.90 (t, *J* = 6.7 Hz, 12H), 1.32 (m, 64H), 1.44 (m, 8H), 1.77 (m, 8H), 2.96 (s, 1H), 3.94 (m, 8H), 5.01 (s, 2H), 5.02 (s, 2H), 5.11 (s, 2H), 6.83 (d, *J* = 2.5 Hz, 1H), 6.86 (d, *J* = 2.5 Hz, 1H), 6.91 (d, *J* = 1.9 Hz, 1H), 6.93 (m, 3H), 6.98 (dd, J_1 = 4.1 Hz, J_2 = 1.8 Hz, 2H), 7.01 (d, *J* = 1.5 Hz, 1H) ppm. **^{13}C-NMR** (100 MHz, $CDCl_3$): δ = 14.46, 23.29, 26.67, 26.70, 29.98, 30.00, 30.09, 30.26, 30.28, 30.31, 30.33, 32.54, 68.52, 69.77, 69.91, 71.75, 71.87, 75.08, 75.26, 114.12, 114.24, 114.26, 115.10, 116.01, 120.84, 120.99, 122.72, 128.30, 130.18, 130.20, 149.63, 149.66, 149.91, 150.04, 153.01 ppm. **IR**: ν = 3268, 2921, 2852, 2121, 1720, 1518, 1267, 1230 cm^{-1}. **MS** ($MALDI^+$) m/z (%): 1132.0 (5.2 %) [M+ Na^+].

3,5-bis((3,4-bis(dodecyloxy)benzyl)oxy)benzyl propiolate (27): White solid. Yield: 50 %. **RMN-^{1}H** (300 MHz, $CDCl_3$): δ = 0.88 (t, *J* = 6.8 Hz, 9H), 1.27 (m, 48H), 1.43 (m, 6H), 1.76 (m, 6H), 2.98 (s, 1H), 3.91 (t, *J* = 6.6 Hz, 2H), 3.96 (t, *J* = 6.6 Hz, 4H), 4.88 (s, 2H), 4.99 (s, 4H), 5.10 (s, 2H), 6.68 (S, 2H), 6.74 (m, 2H), 6.89 (dd, J_1 = 6.8 Hz, J_2 = 1.8 Hz, 4H), 7.23 (d, *J* = 8.5 Hz, 2H), 7.24 (m, 4H),

7.33 (dd, J_1 = 8.4 Hz, J_2 = 1.6 Hz, 4H) ppm. **RMN-^{13}C** (100 MHz, $CDCl_3$): δ = 13.87, 2.69, 26.04, 29.30, 29.32, 29.35, 29.43, 29.45, 29.62, 29.64, 29.67, 31.93, 67.99, 68.09, 70.94, 74.44, 74.65, 74.87, 107.92, 113.96, 114.39, 128.74, 129.35, 129.98, 130.12, 138.47, 152.34, 153.00, 159.01, 159.16 ppm. **IR**: ν = 3264, 2922, 2852, 2118, 1713, 1515, 1246, 1107 cm^{-1}. **MS** (MALDI$^+$) m/z (%): 1053.7 (39.62 %) [$C_{67}H_{98}O_8$, Na]$^+$.

S2.1.4. Chemical characterization of the dendrons.

As a representative example that demonstrates the formation of the alkyne focal point dendron **24** and polybenzyl alcohol precursor **18**, the ^{1}H and ^{13}C NMR spectra are shown in ***Figure S1***. The ^{1}H NMR spectrum (See ***Figure S1a***) clearly shows the signal ***d*** of the methylene protons in alpha position (*-Ar-C<u>H</u>$_2$-OH)* in the alcohol dendron **18** at 4.59 ppm (d, J_1 = 2.5 Hz, 2H). In the next step, when the esterification reaction has occurred, a change in its chemical shift and coupling pattern occurs (See ***Figure S1b***) giving rise to signal ***d*** at 5.11 ppm (s, 2H) in compound **24**. Furthermore, the terminal proton signal of the alkyne ***j*** (*-Ar-CH$_2$-CC<u>H</u>*) appears at 2.99 ppm (s, 1H). In the ^{13}C NMR spectrum, after the esterification reaction of the polybencylic alcohol **18** (***Figure S1c***) to the alkyne focal point dendron **24** (***Figure S1d***) it is possible to observe the chemical shifts of signal ***7*** from 139.2 to 130.9 ppm and of signal ***d*** from 65.6 to 68.6 ppm. Finally, signals ***10***, ***11*** and ***j*** appear at 152.9, 75.5 and 75.0 ppm respectively (See ***Figure S1d***).

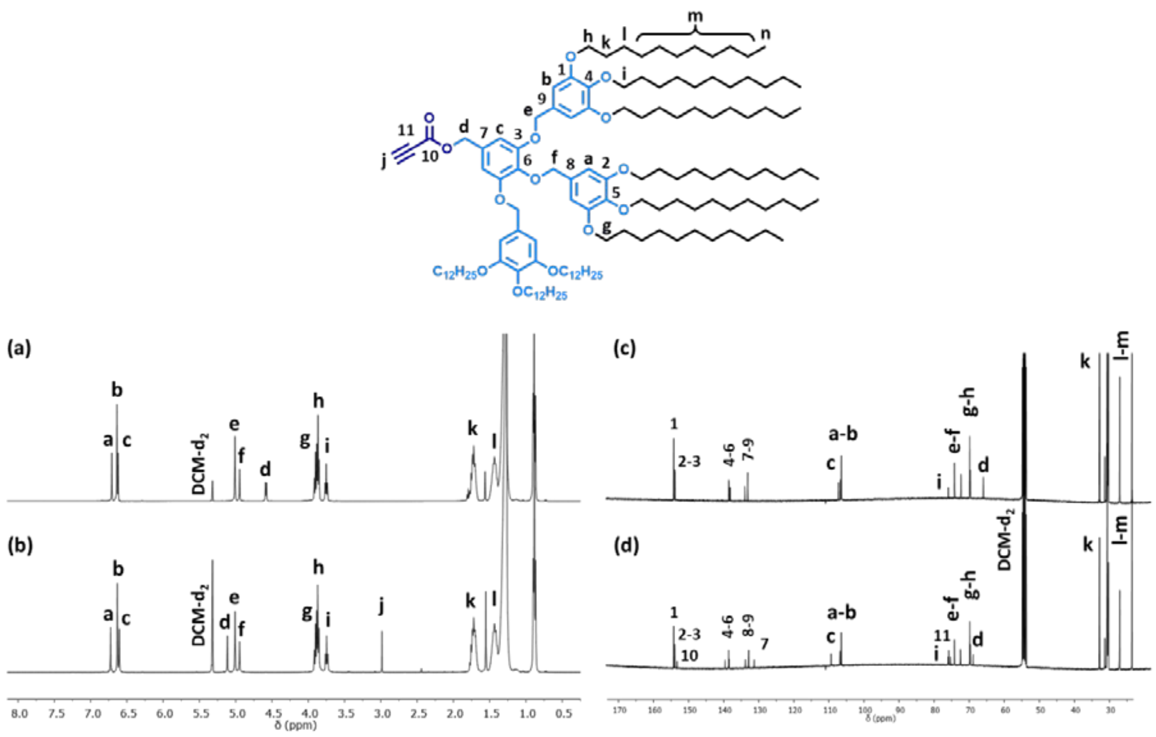

***Figure S1**: ^{1}H (left) and ^{13}C (right) NMR spectra in DCM-d_2 solution at 25 °C of: (**a, c**) polybenzyl alcohol dendron (**18**) and, (**b, d**) alkyne focal point dendron (**24**).*

Attenuated total reflection ATR-FTIR studies were performed and shown in ***Figure S2***. Polybencylic alcohol dendron **18** (***Figure S2a***) shows the typical –OH stretching broad signal at 3250 cm^{-1} and the aromatic C-C bending close to 1500 cm^{-1}. When the formation of the alkyne focal point dendron **24** occurs (***Figure S2b***) the alcohol stretching signal disappears and narrow peaks appears at 3263 and 2117 cm^{-1} corresponding respectively to stretching of the H-C≡ and -C≡C- bonds of the alkyne.

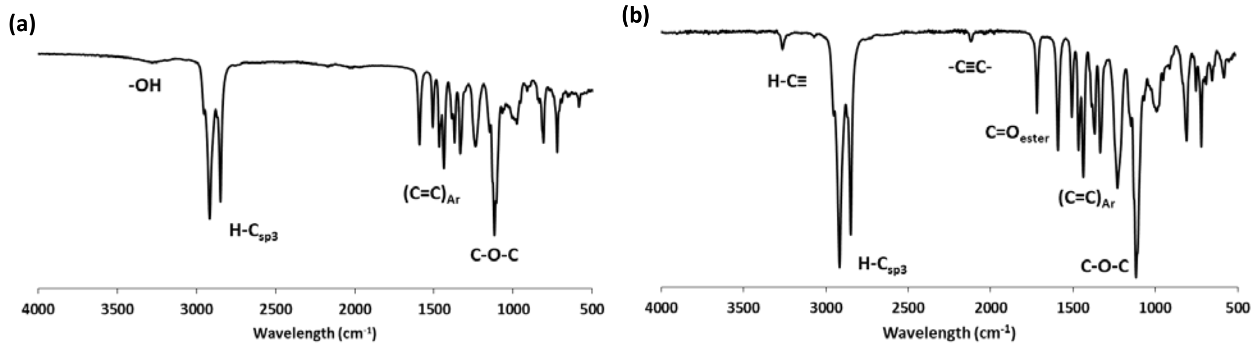

***Figure S2**: ATR-FTIR spectra at 25 ºC of: (**a**) polybenzyl alcohol (**18**) and, (**b**) alkyne focal point dendron (**24**).*

The esterification reaction was also checked by **MS-MALDI** spectrometry by observing the shift in *m/z* values when the coupling occurs. In ***Figure S3*** (top) the polybenzyl alcohol **18** (MW: 2083.8) shows a signal at 2106.8 $[M+Na]^+$. This signal disappears for compound **24** (MW: 2135.8) and a new signal is generated at 2158.2 $[M+Na]^+$ (See ***Figure S3***, bottom).

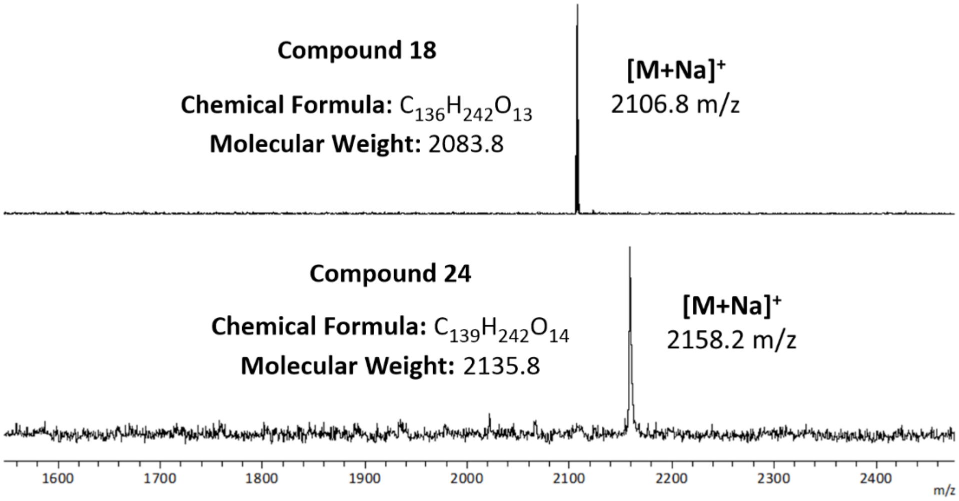

***Figure S3**: MS-MALDI spectra of compounds **18** (top) and **24** (bottom).*

S2.2. Synthesis and characterization of 11-azidoundecane-1-thiol (Compound 30).

In the ***Scheme S4*** is represented the synthetic route of the **11-azidoundecane-1-thiol (30)**

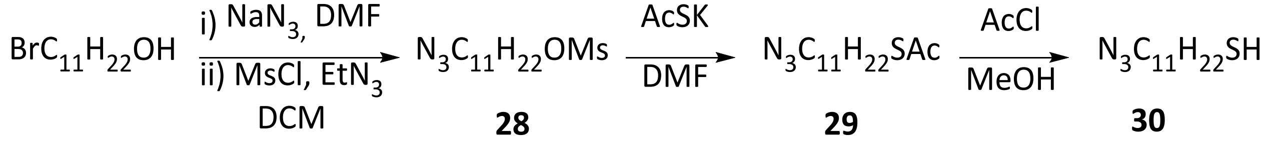

***Scheme S4**: Synthetic route of 11-azidoundecane-1-thiol (**30**).*

Synthesis of 11-azidoundecyl methanesulfonate (28).

In a dry flask under argon atmosphere 11-bromoundecan-1-ol (1 eq.) was dissolved in 30 mL of dry DMF. 2.1 eq. of sodium azide was added and the mixture was stirred and heated at 140 ºC for 24 h. The mixture was allowed to cool to room temperature and then it was poured onto water. The crude product was extracted with ethyl ether (3 times). The organic phase was washed with brine, dried over $MgSO_4$, filtered and the solvent was evaporated by a rotatory evaporator system. The 11-azidoundecan-1-ol obtained (1 eq.) and TEA (3.0 eq) were mixed in dry DCM. The mixture was cooled in a water/ice bath at 0 ºC. Methanesulfonyl chloride (2.9 eq.) was added dropwise and the reaction was stirred overnight at room temperature. $Na_2SO_4·10H_2O$ was added to quench the reaction and then it was poured onto water. The organic phase was washed with HCl 1M, $NaHCO_3$ and brine, dried over $MgSO_4$, filtered and concentrated by rotatory evaporation.

11-azidoundecyl methanesulfonate (28): Yellow oil. Yield: > 95 %. **^{1}H-NMR** (400 MHz, $CDCl_3$): δ = 1.29 (m, 14H), 1.60 (m, 2H), 1.75 (m, 2H), 3.00 (s, 3H), 3.26 (t, *J* = 6.94 Hz, 2H), 4.22 (t, *J* = 6.58 Hz, 2H) ppm. **^{13}C-NMR** (100 MHz, $CDCl_3$): δ = 25.40, 26.69, 28.82, 28.99, 29.10, 29.12, 29.36, 29.39, 37.37, 51.48, 70.17 ppm. **IR**: ν = 2928, 2855, 2096, 1176 cm^{-1}. **MS** ($MALDI^+$) m/z (%): 314.1 (3.12 %) [$M+Na^+$].

Synthesis of S-(11-azidoundecyl) ethanethioate (29).

Compound **28** (1 eq.) was dissolved in dry DMF and cooled in a water/ice bath at 0 ºC. A solution of potassium thioacetate (3.0 eq.) in DMF was added dropwise and the mixture was stirred and heated at 50 ºC overnight. The mixture was allowed to cool to room temperature and then it was poured onto water and extracted with ethyl acetate (AcOEt). The organic phase was washed with brine, dried over $MgSO_4$, filtered and the solvent was evaporated by rotatory evaporator system.

S-(11-azidoundecyl) ethanethioate (29): Yellow oil. Yield: > 95 %. **^{1}H-NMR** (400 MHz, $CDCl_3$): δ = 1.31 (m, 14H), 1.58 (m, 4H), 2.32 (s, 3H), 2.86 (t, *J* = 9.6 Hz, 2H), 3.25 (t, *J* = 6.9 Hz, 2H) ppm. **RMN-^{13}C** (100 MHz, $CDCl_3$): δ = 26.70, 28.79, 28.83, 29.07, 29.11, 29.39, 29.41, 29.48, 30.63, 51.48, 196.03 ppm. **IR**: ν = 2925, 2854, 2095, 1693 cm^{-1}. **MS** (ESI^+) m/z (%): 483.4 (100 %) [2(M-Ac),Na^+].

Synthesis of 11-azidoundecane-1-thiol (30).

The precursory thioester **29** (1 eq.) was dissolved in dry MeOH and cooled in a water/ice bath at 0 °C under argon atmosphere. Acetyl chloride (10 eq.) was added dropwise and the mixture was stirred at room temperature overnight. The organic phase was extracted with ether, washed with brine, dried over $MgSO_4$, filtered and the solvent was evaporated by a rotatory evaporator system.

11-azidoundecane-1-thiol (30): Yellow oil. Yield: > 95 %. **^{1}H-NMR** (300 MHz, DCM-d_2): δ = 1.35 (m, 15H), 1.59 (m, 4H), 2.51 (dd, J_1 = 14.7 Hz, J_2 = 9.4 Hz, 2H), 3.25 (t, *J* = 6.9 Hz, 2H) ppm. **^{13}C-NMR** (100 MHz, $CDCl_3$): δ = 24.67, 26.72, 28.37, 28.52, 28.84, 29.05, 29.14, 29.22, 29.45, 34.04, 51.50 ppm. **IR**: ν = 2926, 2854, 2095, 1466, 1259 cm^{-1}. **MS** (ESI^+) m/z (%): 483.4 (100 %) [2(M-Ac), Na^+].

S2.3 Synthesis and characterization of AuNP.

S2.3.1 Synthesis of gold nanoparticles and ligand exchange reaction (*Scheme S5*).

The gold nanoparticles (**AuDT**) were synthesized by the method of Brust et al. by direct reduction of $HAuCl_4$ with sodium borohydride in the presence of dodecane-1-thiol as stabilizator and TBTA as transfer agent in a biphasic system (toluene and Milli-Q water); the colour change from a yellow to a dark-red dispersion evidenced the nanoparticle formation.

After performing the characterization, 75 mg of **AuDT** were stirred with 50 mg of 11-azidoundecane-1-thiol (**AT**) in DCM (10 mL) as solvent during 48 hours under an argon atmosphere at room temperature to carry out the ligand exchange reaction (scheme S4). The black solid obtained was isolated by rotatory evaporation, dissolved in the minimum amount of DCM and stirred for 20 minutes in 300 mL of EtOH. The dispersion was stored at 20 ^{o}C during 4 hours and a black solid separated itself from the liquid. The solid was isolated by submitting the dispersion to centrifugation at 15000 rpm and, finally, the **AuDT-AT** nanoparticles were dissolved in chloroform and purified by SEC in Sephadex LH-20.

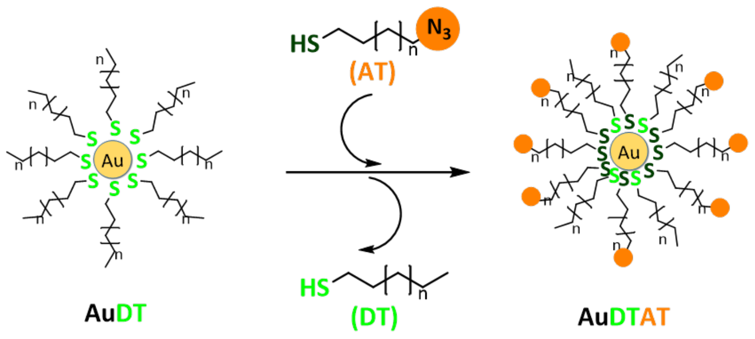

***Scheme S4**: Representation of the ligand exchange reaction of **AuDT** with **11-azidoundecane-1-thiol**.*

In the ***Figure S5*** are gathered the IR spectra of the nanoparticles **AuDT** and **AuDT-AT**. As can be seen in the spectrum of the **AuDT-AT** a sharp band corresponding to the azido group are clearly observed at 2088 cm^{-1}.

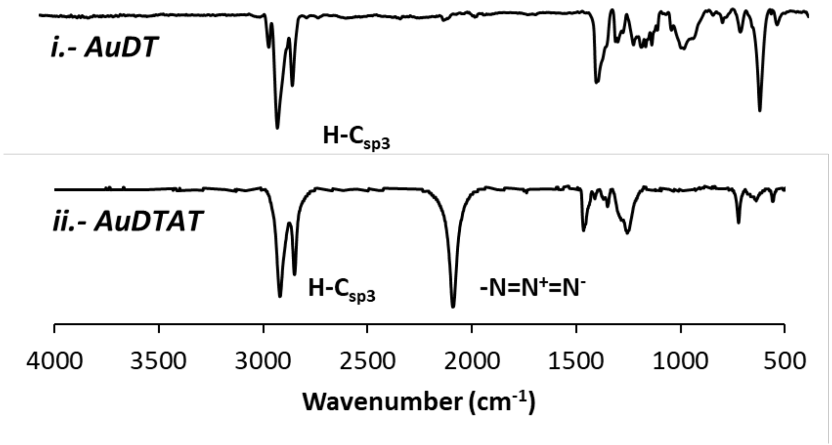

***Figure S5** Infrared spectra of particles (i) **AuDT** and (ii) **AuDT-AT**.*

S2.3.2. Functionalization of 11-azidoundecane-1-thiol gold nanoparticles (AuDT-AT) with alkynyl focal-point dendrons by *click reaction* (*Scheme S6*).

Functionalization of AuDT-AT nanoparticles with alkynyl dendritic ligands by Huisgen 1,3-dipolar cycloadition.

In a Schlenk flask, 50 mg of **AuDT-AT** and 66 µmol of dendritic ligand were mixed in dry THF and vacuum/argon cycles (3 times) were applied. The reaction was stirred for 20 min at 30 °C. In another flask, $CuSO_4 \cdot 5H_2O$ (0.10 eq), TBTA (0.10 eq) and sodium *L*-ascorbate (0.2 eq) are mixed in DMF (2 mL) and H_2O (40 µL) as solvents. The mixture was stirred under argon atmosphere at 30 °C. The change of coloration from blue to pale-yellow evidenced the copper reduction (from

Cu^{II} to Cu^{I}) and stabilization (TBTA-Cu^{I} complex). The copper complex was added to the Schlenk flask that contains **AuDT-AT** and the dendritic ligand, and the mixture was stirred for 48 h under an argon atmosphere at 30 °C. The black crude product obtained was washed three times with a DCM:NH_4OH 15N mixture. The organic phase was dried over $MgSO_4$, filtered and the solvent was removed by rotary evaporation. Finally, the resukting **AuNP** were dissolved in chloroform and purified by size exclusion chromatography in Sephadex LH-20.

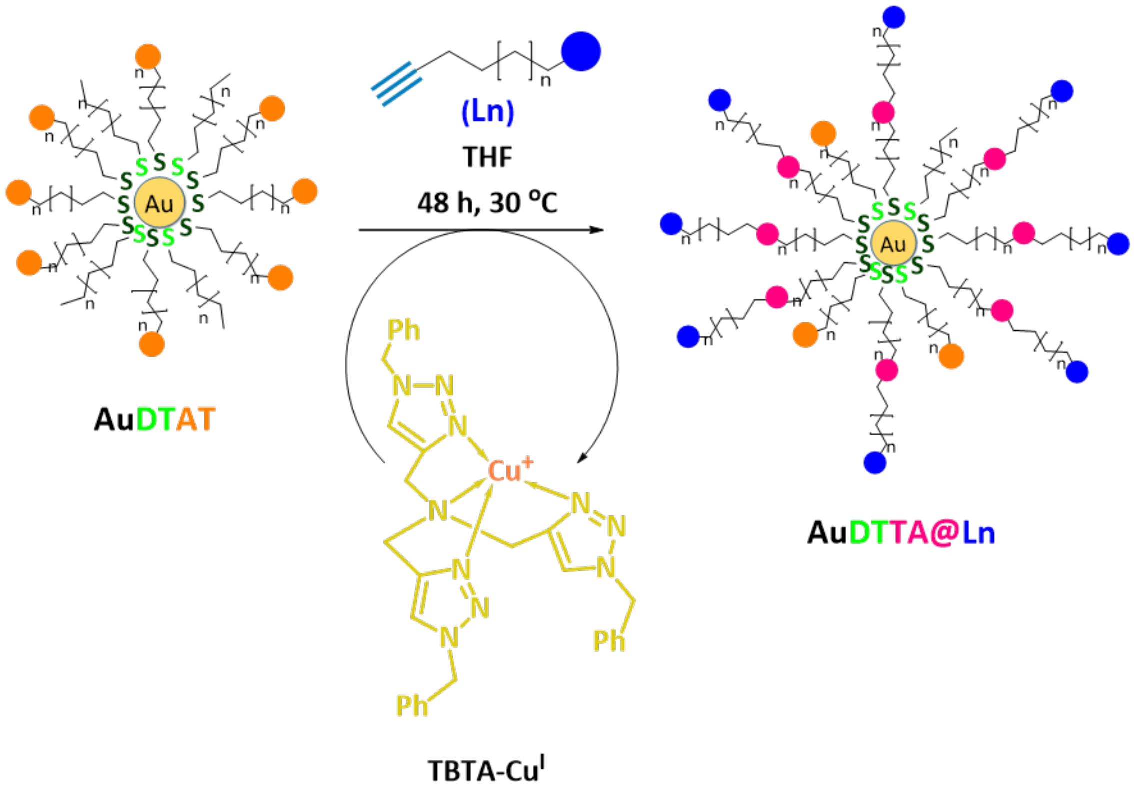

***Scheme S6**: Representation of azide-alkyne Huisgen cycloaddition of **AuDT-AT** and **Ln** dendrons*

S3.Mesogenic and optical characterization of the alkynyl focal-point dendrimers.

S3.1 Thermogravimetric analysis of alkynyl focal-point dendrons.

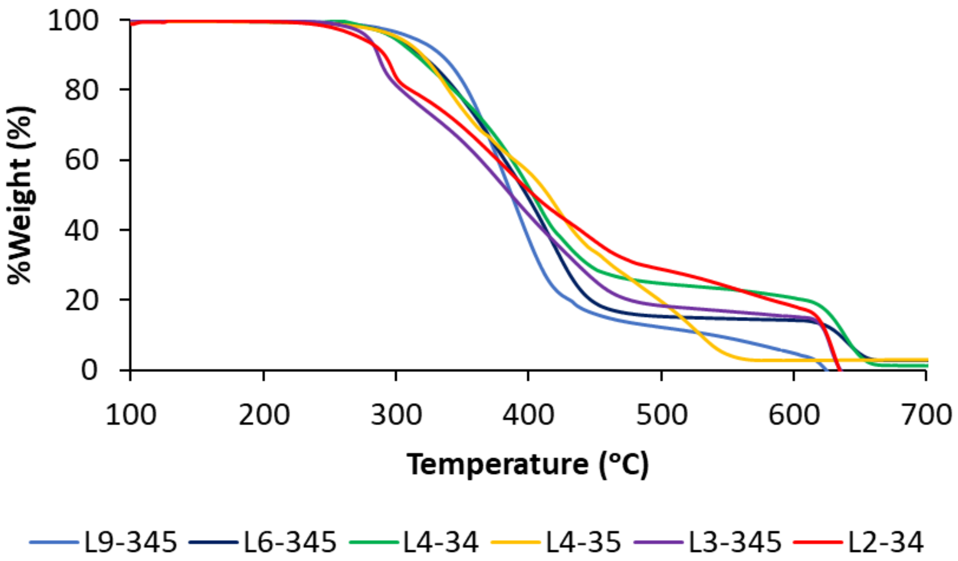

Figure S7: *TGA thermograms of the alkynyl focal point dendrons.*

S3.2. Optical textures of alkynyl focal-point dendrons.

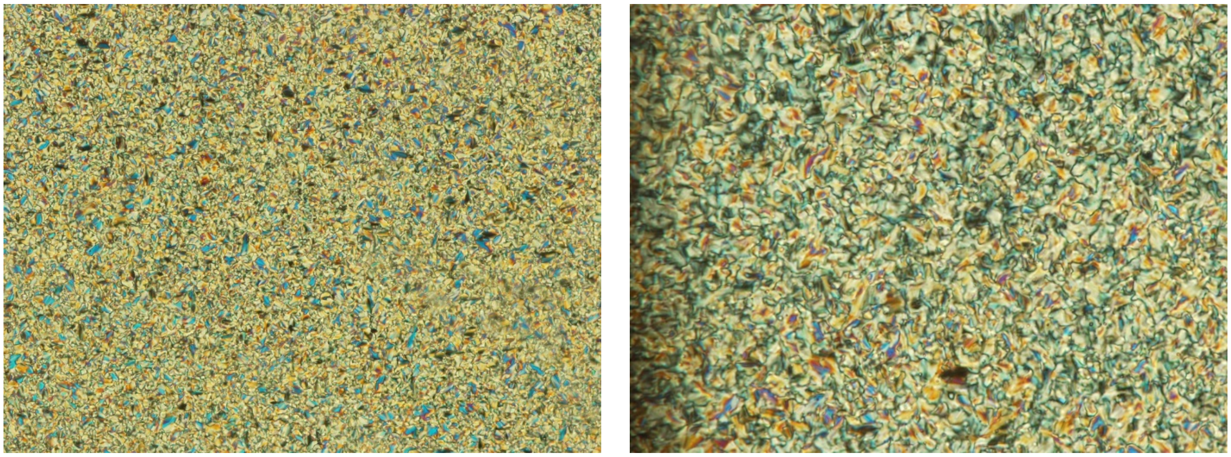

(a) (b)

Figure S8: *Optical textures of (a) compound 1(L2-3,4) at 56 °C in the cooling process (x50 magnification); (b) compound 4(L3-3,4,5) at 40 °C in the cooling process (x50 magnification).*

S3.3. XRD studies of alkynyl focal-point dendrons.

Table S1: *X-Ray diffraction data of alkyne focal-point ligands.*

Compound	Phase[a]	T °C	Structural parameters[b] Å	d_{obs}[c] Å	h k l[d]
**L2-34**	Col_r	55	*a* = 128; b =52; *h* = 4.8; Z/2 = 12	63.6	200
				48.1	110
				32.3	400
				26.0	020
				24.3	220
**L3-345**	Col_h	RT	*a* = 48; *h* = 5.0; Z = 6	42.1	100
				23.9	110
				20.5	200
				15.7	210
**L4-34**	Col_h	RT	*a* = 58; *h* = 4.9; Z = 8	50.7	100
				29.3	110
				24.7	200
**L6-345**	Col_h	RT	*a* = 50; *h* = 5.0; Z =4	42.7	100
				26.2	110
				21.1	200

[a] Col_h: hexagonal columnar mesophase; Col_r: rectangular columnar rectangular mesophase; Col_x: undetermined columnar mesophase; I: Isotropic liquid and, C: Crystal phase. [b] Structural parameters obtained by XRD. *a*, *b*: lattice constants of the columnar phases (Å), *h*: estimated mean stacking distance (Å), Z: number of molecules per disk. [c] d_{obs}: spacing calculated from each X-ray maxima by applying Bragg`s law. [d] Miller indices.

From the diffractograms it was concluded that the mesophases exhibited by the alkynyl dendrons are columnar hexagonal (Col_h), except for the columnar mesophase of **L2-3,4**, which has a rectangular symmetry (Col_r). It was not possible to study the columnar mesophase of **L9-3,4,5** due to its monotropic nature and concomitant tendency to crystallization.

The following relationship exists between the unit cell volume V, the density in the mesophase ρ and the number of molecules per unit cell Z:

$$\rho = \frac{M \cdot Z}{N_A \cdot V}$$

where M is the molar mass (in g mol^{-1}), N_A Avogadro's number and V the cell volume in cm^3.

For a hexagonal columnar (Col_h) mesophase there is one column per cell. However, a rectangular columnar (Col_r) mesophase usually contains two columns per cell, and therefore the number of molecules per disk is Z/2. The number of dendritic molecules necessary to fill the cross-section of a column was calculated from the previous equation. Parameter *c* of the unit cell is unknown because there is not regular intracolumnar order and therefore a scattering maximum corresponding to the inter-disk stacking distance is absent. However, even if the mean stacking distance is unknown, it is possible to estimate the number of molecules necessary to fill a column slice of a given thickness. Assuming a density close to 1 g/cm^{-3}, the Z values for each columnar mesophase (or Z/2 for the Col_r mesophase of **L2-3,4**) can be estimated and their values are gathered in Table 1 for a column slice 4.8-5.0 Å thick. From these values it is deduced that the bigger the dendritic molecule, the lower number of molecules necessary to generate a disk. A schematic representation of this aggregation is shown in ***Figure S6***.

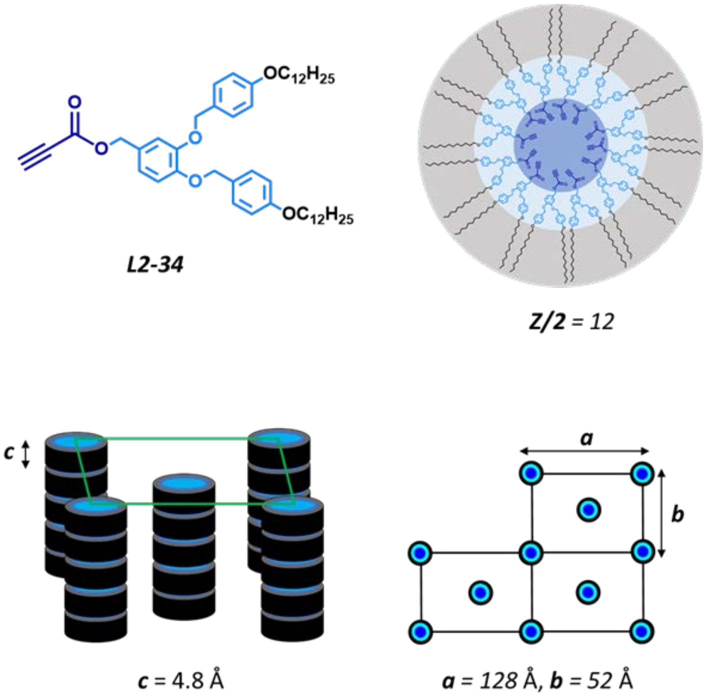

Figure S9: *Representation of the two-dimensional packing of columns for dendron **L2-3,4** in its rectangular columnar mesophase (**Col_r**).*

S3.4. UV-Vis studies of alkynyl focal-point dendrons.

Table S2: *UV-Vis absorption and emission data of alkyne-focal point dendrons.*

Compounds	$\lambda_{max, abs}$	Molar coefficient, ε	$\lambda_{max, emi}$	Emission intensity
	nm	$M^{-1}\cdot cm^{-1}$	nm	a.u.
**L2-34**	279	3750	409	22
	327	850	-	-
**L3-345**	275	3150	405	15
**L4-34**	283	9050	398	12
**L4-35**	283	6700	405	14
**L6-345**	282	6700	410	17
**L9-345**	274	3250	407	31

All the values are obtained from 10^{-5} M solutions in chloroform.

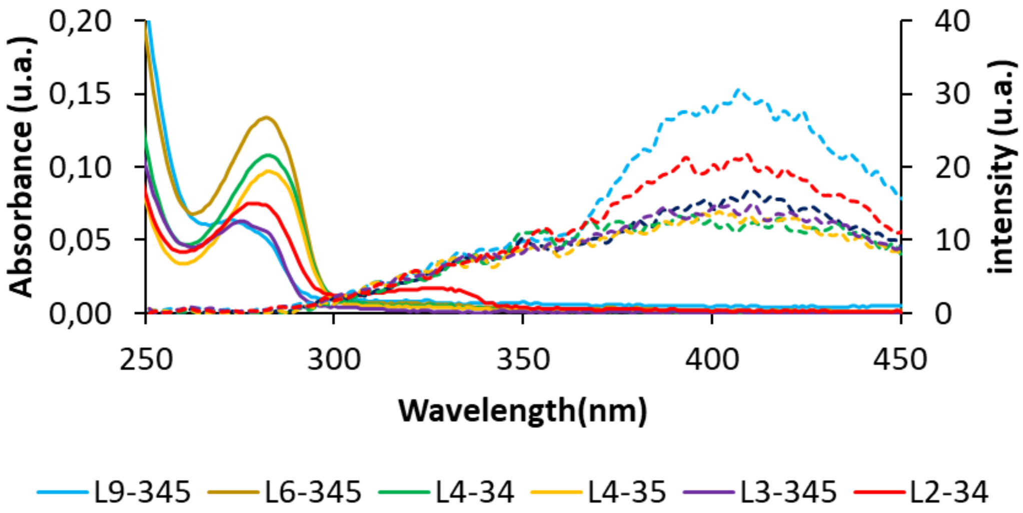

***Figure S10**: **UV-Vis** absorption spectra (continuous lines, absorbance in arbitrary units, u.a.) and fluorescence emission (dashed lines, intensity in u.a.) for the alkynyl dendrons. HPLC-grade DCM was used as the solvent in both studies.*

S4. Structural characterization of the AuDT-TA@Ln nanoparticles.

S4.1. ^{1}H-NMR spectra of *AuDT*, *AuDT-AT* and *AuDT-TA@Ln* gold nanoparticles.

In ***Figures S10-S17*** are gathered the ^{1}**H-NMR** spectra of the nanoparticles synthesized. All the spectra were collected in DCM-d_2 solution at 25 ºC.

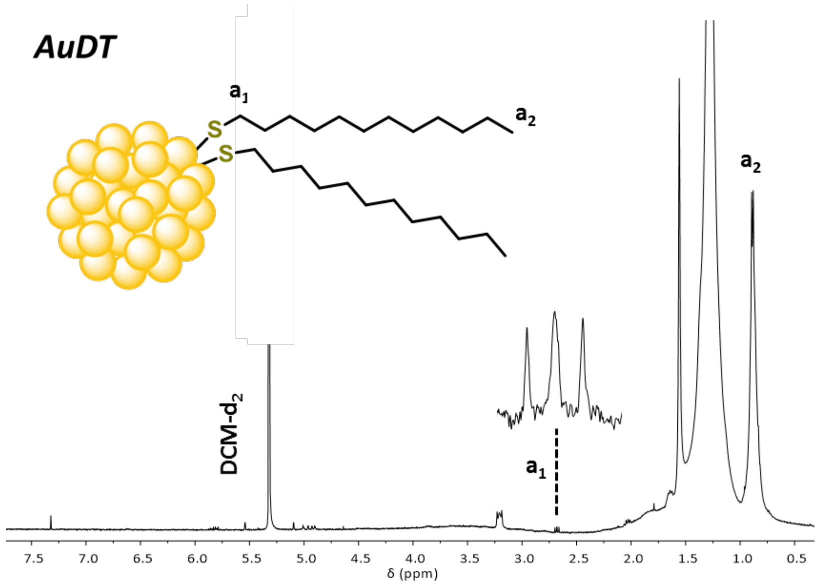

Figure S11: *^{1}**H NMR** spectrum of **AuDT** in DCM-d_2 solution at 25 ºC.*

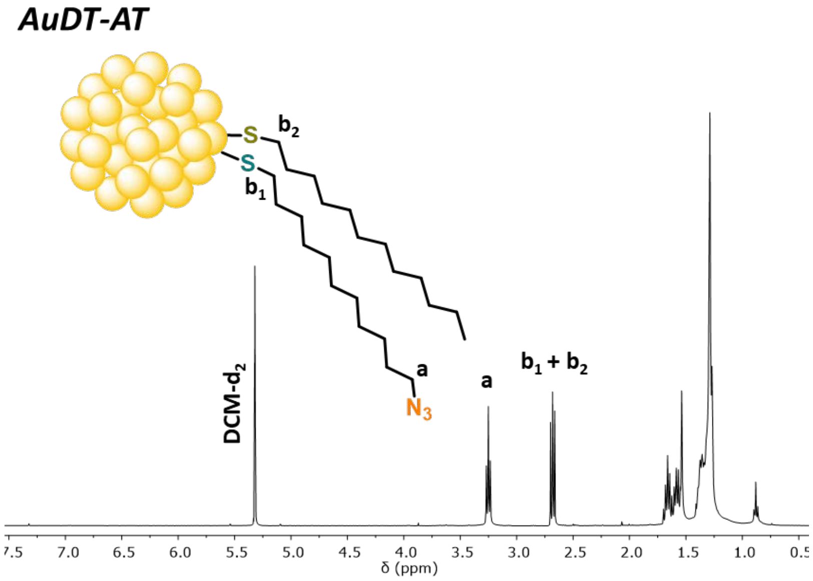

Figure S12: *^{1}**H NMR** spectrum of **AuDT-AT** in DCM-d_2 solution at 25 ºC.*

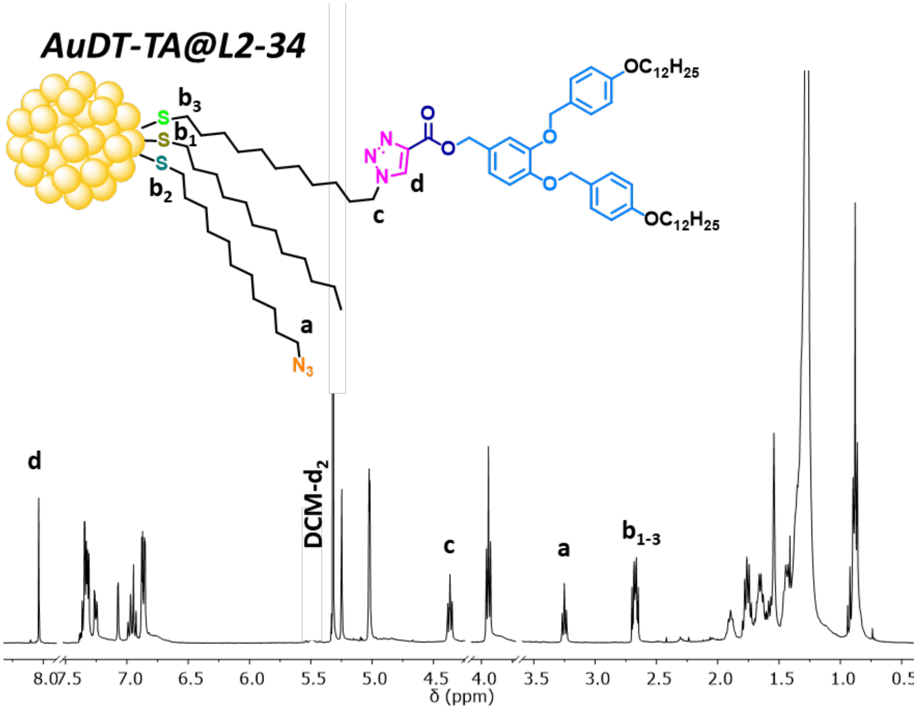

Figure S13: ***^{1}H NMR*** *spectrum of* ***AuDT-TA@L2-3,4*** *in DCM-d_2 solution at 25 ºC.*

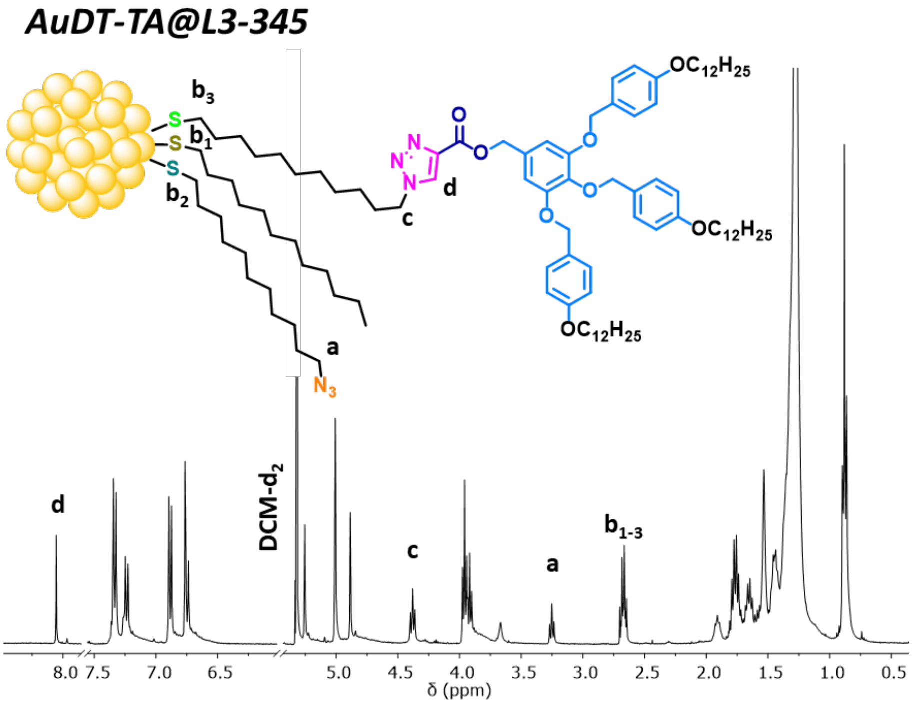

Figure S14: ***^{1}H NMR*** *spectrum of* ***AuDT-TA@L3-3,4,5*** *in DCM-d_2 solution at 25 ºC.*

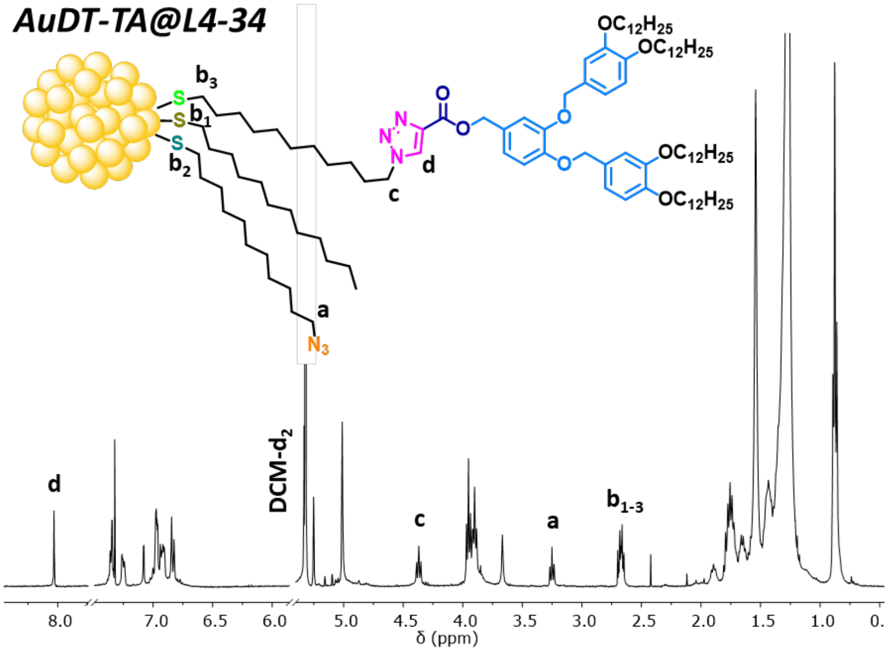

Figure S15: ***^{1}H NMR*** *spectrum of* ***AuDT-TA@L4-3,4*** *in DCM-d_2 solution at 25 ºC.*

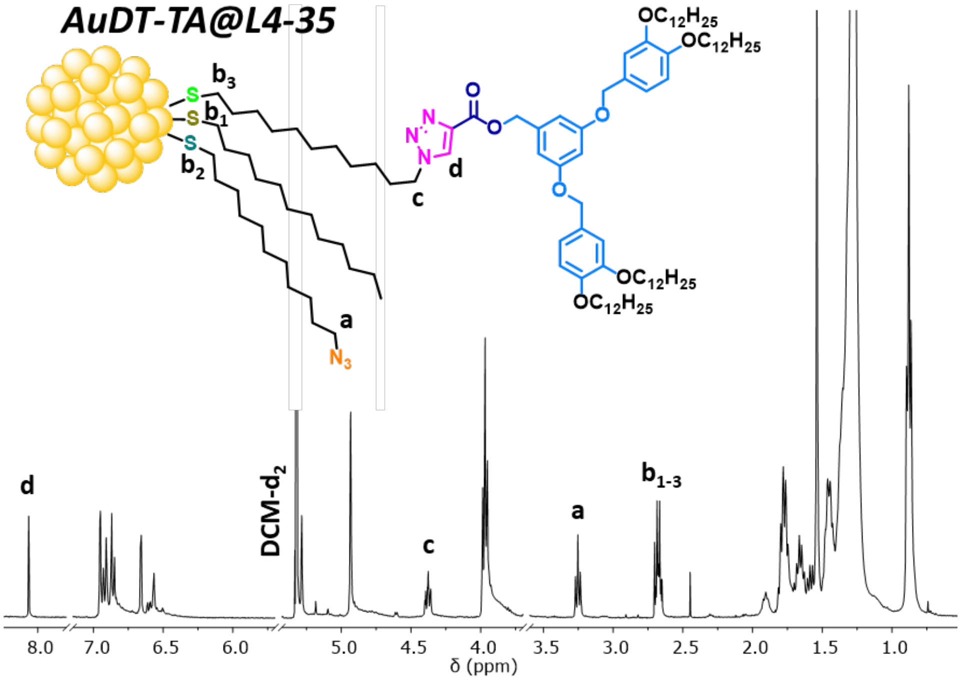

Figure S16: ***^{1}H NMR*** *spectrum of* ***AuDT-TA@L4-3,5*** *in DCM-d_2 solution at 25 ºC.*

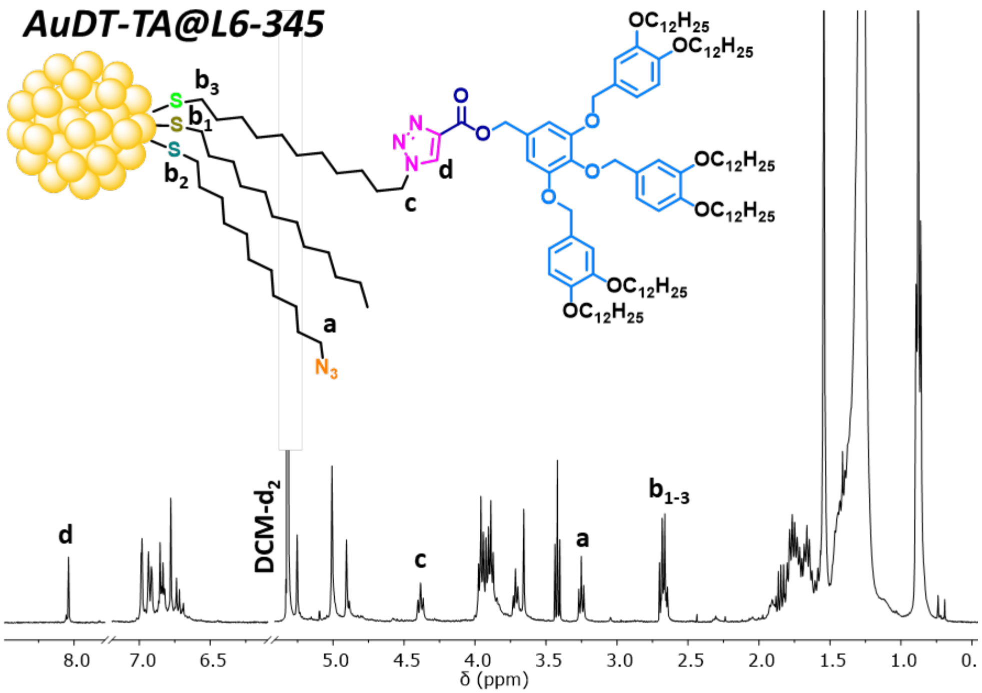

Figure S17: ***^{1}H NMR*** *spectrum of* ***AuDT -TA@L6-3,4,5*** *in DCM-d$_2$ solution at 25 °C.*

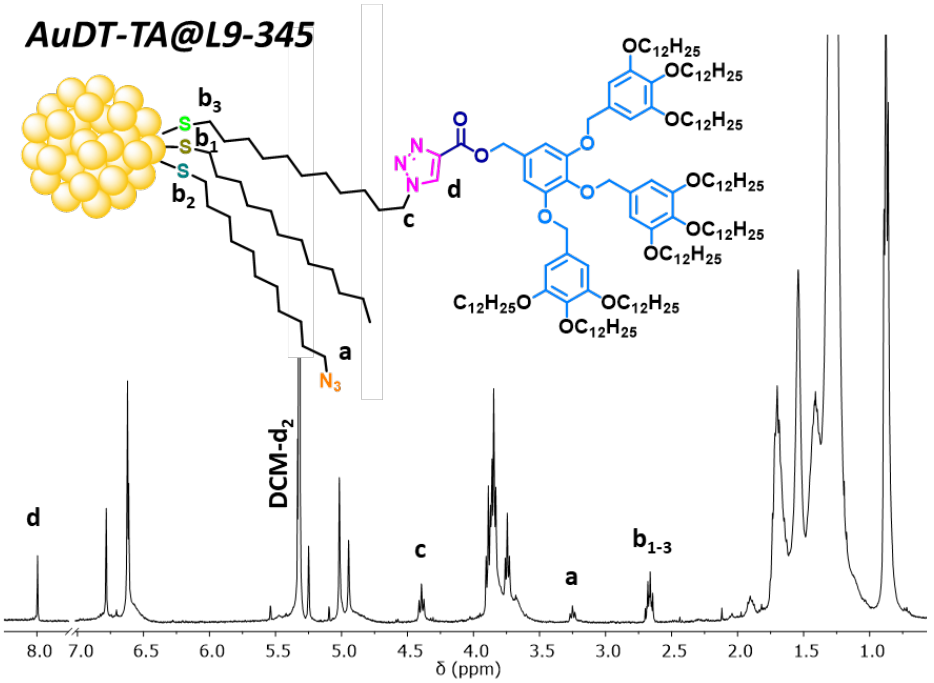

Figure S18: ***^{1}H NMR*** *spectrum of* ***AuDT-TA@L9-3,4,5*** *in DCM-d$_2$ solution at 25 °C.*

S4.2. UV-Vis studies of *AuDT-TA@Ln*

Table S3: *Absorption and emission values of the* ***AuDT-AT-TA@Ln*** *nanoparticles in DCM solution.*

Muestra	$\lambda_{max, abs}$ nm	Absorbancia u.a.	$\lambda_{max, em}$ nm	Intensidad u.a.
**AuDT-TA@L2-34**	277	0.474	439	10
**AuDT-TA@L3-345**	275	0.588	436	7
**AuDT-TA@L4-34**	283	0.623	441	28
**AuDT-TA@L4-35**	283	0.458	438	31
**AuDT-TA@L6-345**	282	0.546	440	20
**AuDT-TA@L9-345**	271	0.321	444	11

Absorption and emission values in nm. Absorbance and emission intensity in arbitrary units (u.a.). Studies carried out in HPLC-quality DCM as solvent.

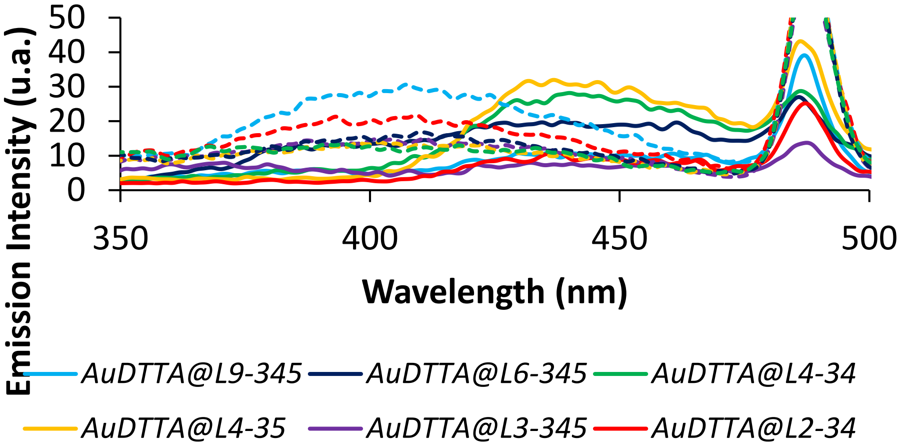

Figure S19: *Fluorescence emission spectra of alkynyl dendrons (dashed lines) and dendrons incorporated into NP´s by cycloaddition (solid lines). HPLC-grade DCM was used as the solvent.*

S4.3. XPS studies of *AuDT-TA@Ln*

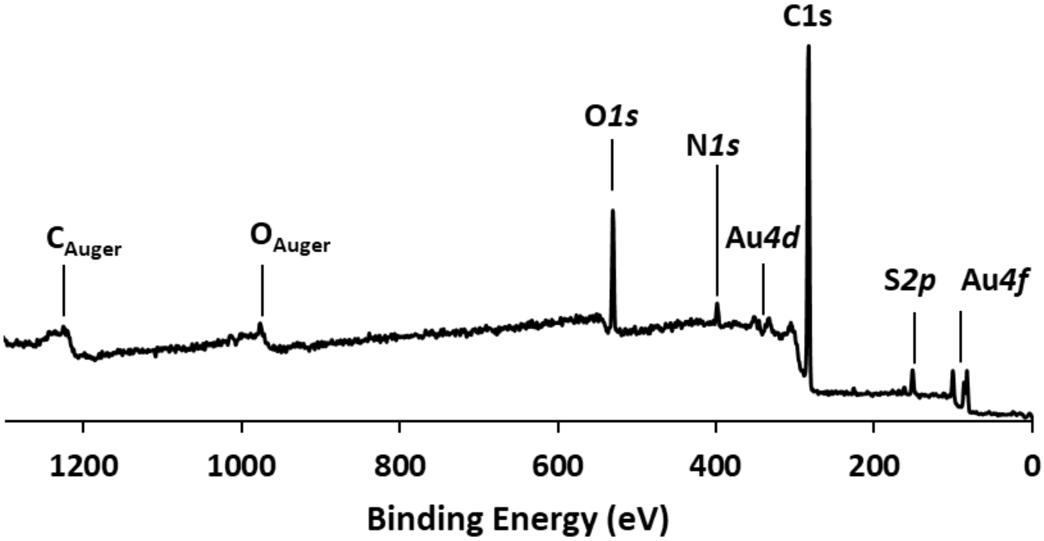

Figure S20: Survey *XPS spectrum of* ***AuDT-TA@L3-3,4,5*** at 0 min treatment thermic time.

Table S4: *XPS data of* ***AuDT-TA@Ln****.*

Sample	Time	S $2p_{3/2}$	S $2p_{1/2}$	Au $4f_{7/2}$	Au $4f_{5/2}$
	min	eV	eV	eV	eV
**AuDT-AT**	0	162.1	163.3	84.1	87.8
**AuDT-TA@L2-3,4**	0	162.6	163.8	84.3	88.0
	180	163.0	164.4	83.9	87.5
**AuDT-TA@L3-3,4,5**	0	161.8	163.0	84.0	87.6
	180	163.0	164.4	84.0	87.8
**AuDT-TA@L4-3,4**	0	163.7	164.9	84.4	88.0
	180	163.2	164.3	84.1	87.4
**AuDT-TA@L6-3,4,5**	0	164.0	165.2	NSO	NSO
	180	162.8	163.9	84.2	87.9
**AuDT-TA@L9-3,4,5**	0	162.1	163.3	84.7	87.9
	180	163.1	164.2	83.8	87.4

HR-XPS values (in eV) for Sulphur (S2p $_{3/2}$ and S2p $_{½}$ orbitals) and, gold (Au4f $_{7/2}$ y Au4f $_{5/2}$ orbitals) of ***AuDT-TA@Ln*** samples. NSO means no signal observed.

S5. Characterization of the gold nanoparticles after the isothermal treatment.

S5.1. TEM exploratory study of the isothermal treatment of the *AuDT-TA@L2-3,4* nanoparticles.

When the thermally-treated nanoparticles were studied by TEM, no evident signs of aggregation or decomposition were observed. The results of this study are collected in the ***Table S5*** and ***Figures S18*** and ***S19***. After 180 min of thermal treatment, the data show a small growth at 120 °C, whereas at 180 °C growth occurs to a larger extent and at a higher rate. From these data it can be deduced that there is a direct relationship between the growth of the nanoparticles and the temperature, in such a way that the higher the treatment temperature the more effective the growth.

Table S5: TEM *data of* ***AuDT-TA@L2-3,4*** *at 120. 150 and 180 °C.*

Sample	Temperature	Time	ϕ	SD
	°C	min	nm	nm
**AuDT-TA@L2-3,4**	120	0	2.2	0.3
		30	2.0	0.4
		60	2.2	0.4
		120	5.6	1.7
		180	5.5	1.0
	150	0	2.2	0.3
		30	2.5	0.7
		60	2.5	1.1
		120	7.0	0.9
		180	12.5	1.3
	180	0	2.2	0.3
		30	5.4	1.4
		60	12.0	2.2
		120	14.1	3.1
		180	15.7	3.0

Temperature (°C), time of treatment (min), mean diameter ϕ and statistical dispersion SD (nm) measured by **TEM**

Moreover, after 180 min of thermal treatment, the TEM images (***Figure S18***) show a moderate size polydispersity for the isotherm at 120 °C, whereas the thermal treatment at 180 °C generates a high polydispersity. On the other hand, the results obtained after the treatment at 150 °C are acceptable in terms of both size and dispersity ***(Figure S19)***. Thus, it is concluded that for an efficient growth without a significant loss of monodispersity in size or shape an isotherm at an intermediate temperature of 150 °C represents the most appropriate balance.

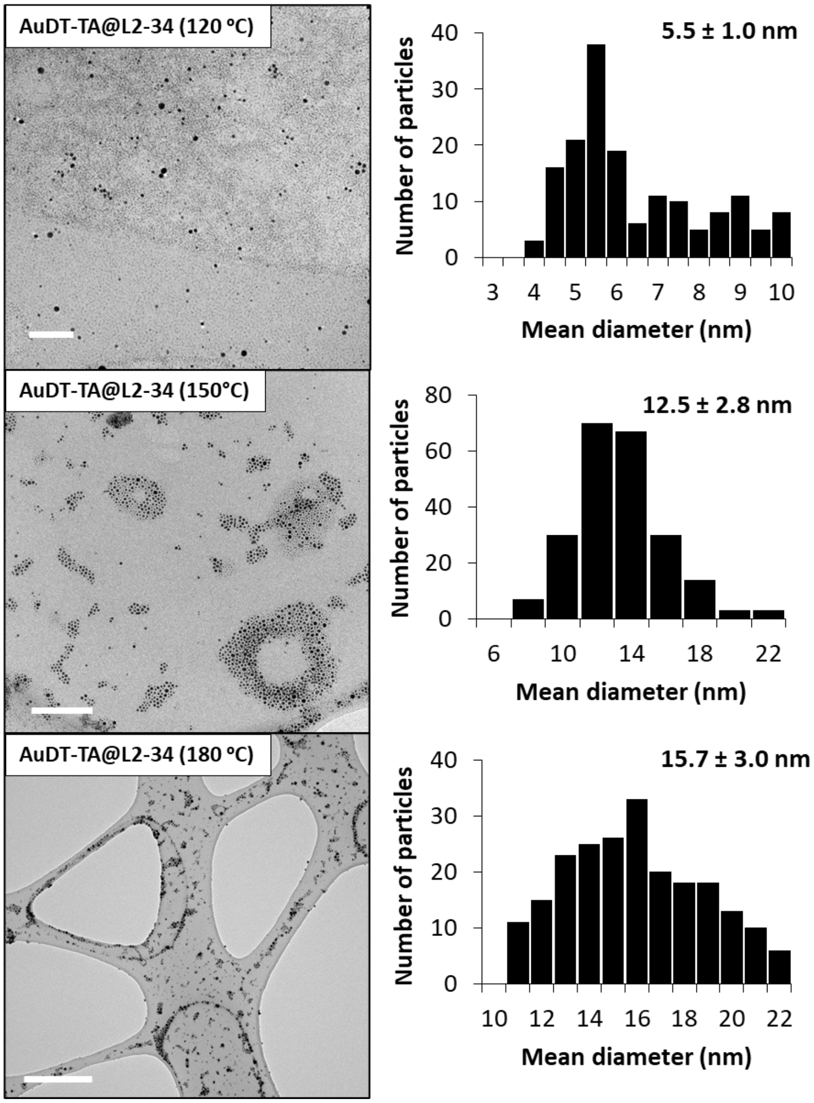

Figure S21: *Photographs and histograms obtained by* ***TEM*** *at 200 kV for* ***AuDT-TA@L2-3,4*** *samples after 180 minutes of treatment at 120 ºC (100 nm scale) and 180 ºC (500 nm scale). The* ***TEM*** *photographs and histograms obtained after treatment at 150 ºC are included in* ***Figure 5*** *of the main text.*

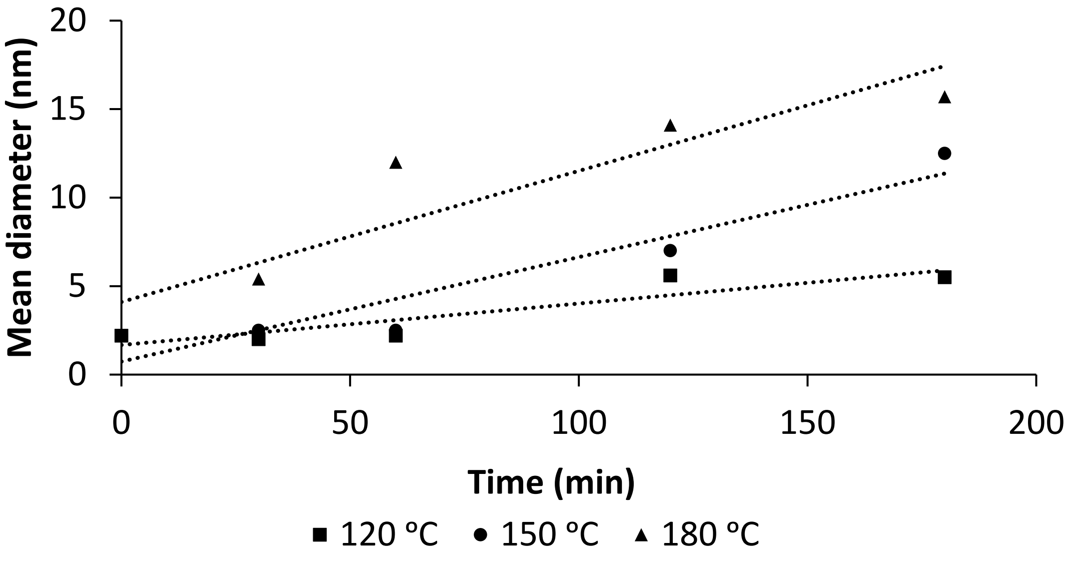

Figure S22: *Growths of the nanoparticle mean diameters ϕ (in nm) versus the reaction time (in min) at isotherms of 120, 150 and 180 °C for the **AuDT-TA@L2-3,4** particles obtained by cycloaddition.*

S5.2. TEM characterization of the gold nanoparticles after a thermal treatment at 150 °C for 0, 30, 60, 120 and 180 minutes.

Table S6: TEM** data of **AuNP`s

Sample	Time	ϕ	SD	a_i
	min	nm	nm	Å
**AuDT-AT**	0	2.2	0.4	-
	0	2.2	0.3	-
	30	2.5	0.4	4.0
**AuDT-TA@L2-3,4**	60	2.5	0.4	4.1
	120	7.0	1.9	4.1
	180	12.5	2.8	4.1
	0	2.1	0.3	-
	30	4.2	0.7	4.1
**AuDT-TA@L3-3,4,5**	60	5.7	1.1	4.1
	120	6.0	0.9	4.1
	180	6.3	1.3	4.1
	0	2.2	0.3	-
	30	4.9	0.8	4.1
**AuDT-TA@L4-3,4**	60	5.3	1.0	4.1
	120	8.9	1.6	4.1
	180	9.1	1.5	4.1
	0	2.0	0.3	-
	30	6.6	2.6	4.1
**AuDT-TA@L6-3,4,5**	60	8.7	2.4	4.1
	120	11.3	2.5	4.1
	180	11.7	2.2	4.1
	0	2.0	0.3	-
	30	3.1	0.8	4.1
**AuDT-TA@L9-3,4,5**	60	5.3	1.3	4.0
	120	7.1	1.4	4.1
	180	7.6	1.4	4.1

Time of treatment (min), mean diameter ϕ and statistical dispersion SD (nm) measured by **TEM**, and cell parameter a_i measured by **SAED** of ***AuDT-TA@Ln*** nanoparticles obtained after thermal treatment.

S5.3. Selected area electron diffraction (SAED) studies of *AuDT-TA@Ln.*

***Table S7: SAED** data of **AuDT-TA@L2-3,4** at different temperatures.*

Sample	Time<br>min	a_i<br>Å	d<br>nm	h	k	l
AuDT-TA@L2-3,4	30	4.1	0.225	1	1	1
			0.123	3	1	1
	60	4.1	0.240	1	1	1
			0.207	2	0	0
			0.144	2	2	0
			0.123	3	1	1
	120	4.1	0.239	1	1	1
			0.206	2	0	0
			0.147	2	2	0
			0.123	3	1	1
	180	4.1	0.239	1	1	1
			0.208	2	0	0
			0.147	2	2	0
			0.124	3	1	1

Structural parameters obtained: cell constant a_i (Å), d_{obs} calculated according to Bragg's law, and Miller Indices.

***Table S8: SAED** data of **AuDT-TA@L3-3,4,5** at different temperatures.*

Sample	Time<br>min	a_i<br>Å	d<br>nm	h	k	l
AuDT-TA@L3-3,4,5	30	4.1	0.240	1	1	1
			0.205	2	0	0
			0.146	2	2	0
			0.124	3	1	1
	60	4.1	0.239	1	1	1
			0.206	2	0	0
			0.147	2	2	0
			0.125	3	1	1
	120	4.1	0.234	1	1	1
			0.203	2	0	0
			0.145	2	2	0
			0.124	3	1	1
	180	4.1	0.236	1	1	1
			0.204	2	0	0
			0.147	2	2	0
			0.125	3	1	1

Structural parameters obtained: cell constant a_i (Å), d_{obs} calculated according to Bragg's law, and Miller Indices.

***Table S9: SAED** data of **AuDT-TA@L4-3,4** at different temperatures.*

Sample	Time<br>min	a_i<br>Å	d<br>nm	h	k	l
**AuDT-TA@L4-3,4**	30	4.1	0.235	1	1	1
			0.207	2	0	0
			0.146	2	2	0
			0.124	3	1	1
	60	4.1	0.233	1	1	1
			0.203	2	0	0
			0.147	2	2	0
			0.124	3	1	1
	120	4.1	0.238	1	1	1
			0.204	2	0	0
			0.147	2	2	0
			0.125	3	1	1
	180	4.1	0.234	1	1	1
			0.207	2	0	0
			0.147	2	2	0
			0.124	3	1	1

Structural parameters obtained: cell constant a_i (Å), d_{obs} calculated according to Bragg's law, and Miller Indices.

***Table S10: SAED** data of **AuDT-TA@L6-3,4,5** at different temperatures.*

Sample	Time<br>min	a_i<br>Å	d<br>nm	h	k	l
**AuDT-TA@L6-3,4,5**	30	4.1	0.236	1	1	1
			0.203	2	0	0
			0.145	2	2	0
			0.125	3	1	1
	60	4.1	0.233	1	1	1
			0.205	2	0	0
			0.146	2	2	0
			0.124	3	1	1
	120	4.1	0.235	1	1	1
			0.205	2	0	0
			0.146	2	2	0
			0.125	3	1	1
	180	4.1	0.240	1	1	1
			0.207	2	0	0
			0.148	2	2	0
			0.123	3	1	1

Structural parameters obtained: cell constant a_i (Å), d_{obs} calculated according to Bragg's law, and Miller Indices.

***Table S11: SAED** data of **AuDT-TA@L9-3,4,5** at different temperatures.*

**Sample**	**Time** min	**a_i** Å	**d** nm	**h**	**k**	**l**
**AuDT-TA@L9-3,4,5**	30	4.1	0.236	1	1	1
			0.146	2	2	0
			0.124	3	1	1
	60	4.1	0.234	1	1	1
			0.201	2	0	0
			0.144	2	2	0
			0.122	3	1	1
	120	4.1	0.239	1	1	1
			0.209	2	0	0
			0.145	2	2	0
			0.122	3	1	1
	180	4.1	0.240	1	1	1
			0.206	2	0	0
			0.145	2	2	0
			0.122	3	1	1

Structural parameters obtained: cell constant a_i (Å), d_{obs} calculated according to Bragg's law, and Miller Indices.

S5.4. Ligand percentages in the *AuDT-TA@Ln* nanoparticles.

In Table S12 are gathered the ligand percentages in the gold nanoparticles obtained from the area of the signals observed in the [1]*HNMR studies.*

Table S12: *Area values and ligand percentage for* ***AuDT-TA@Ln****.*

Sample	t<br>min	$A_{I\ (TA)}$	$A_{II\ (AT)}$	$A_{III\ (DT)}$	% $L_{I\ (TA)}$<br>%	% $L_{II\ (AT)}$<br>%	% $L_{III\ (DT)}$<br>%
**AuDT-TA@L2-3,4**	0	2826	2089	1017	47.6	35.2	17.1
	60	1154	713	655	45.8	28.3	26.0
	120	1528	1045	720	46.4	31.7	21.9
	180	1372	883	608	47.9	30.8	21.2
**AuDT-TA@L3-3,4,5**	0	2589	1697	922	49.7	32.6	17.7
	60	2553	1285	1351	49.2	24.8	26.0
	120	2459	1241	1284	49.3	24.9	25.8
	180	1994	966	963	50.8	24.6	24.5
**AuDT-TA@L4-3,4**	0	1327	1014	536	46.1	35.2	18.6
	60	2765	2138	1502	43.2	33.4	23.5
	120	1445	905	723	47.0	29.5	23.5
	180	2231	1650	1315	42.9	31.8	25.3
**AuDT-TA@L6-3,4,5**	0	770	434	449	46.6	26.2	27.2
	60	618	0	648	48.8	0.0	51.2
	120	785	0	865	47.6	0.0	52.4
	180	737	0	807	47.7	0.0	52.3
**AuDT-TA@L9-3,4,5**	0	771	340	441	49.7	21.9	28.4
	60	687	379	435	45.8	25.2	29.0
	120	1069	600	803	43.2	24.3	32.5
	180	1099	532	753	46.1	22.3	31.6

Reaction time (min), areas measured from ^{1}**H-NMR** spectra. The proportion of each ligand [% L_{DT}, % L_{AT} and % L_n] (% molar) was obtained by means of a one-dimensional quantitative experiment of ^{1}**H NMR**.

***S5.5. Table S13: UV-Vis** data of **AuDT-TA@Ln**.*

**Sample**	**Time**	**ϕ**	**$\lambda_{solution}$**	**λ_{solid}**
	min	nm	nm	nm
**AuDT-TA@L2-3,4**	30	2.5	516	NSO
	60	2.5	516	562
	120	7.0	522	565
	180	12.5	533	570
**AuDT-TA@L3-3,4,5**	30	4.2	519	NSO
	60	5.7	530	531
	120	6.0	532	537
	180	6.3	532	544
**AuDT-TA@L4-3,4**	30	4.9	519	NSO
	60	5.3	528	573
	120	8.9	529	581
	180	9.1	545	593
**AuDT-TA@L6-3,4,5**	30	6.6	530	538
	60	8.7	530	555
	120	11.3	531	563
	180	11.7	532	566
**AuDT-TA@L9-3,4,5**	30	3.1	518	524
	60	5.3	525	533
	120	7.1	530	548
	180	7.6	530	548

Time of treatment (min) and mean diameter ϕ obtained by **TEM**, $\lambda_{solution}$ and λ_{solid} (nm) of samples of ***AuDT-TA@Ln*** in DCM solution. The solid was obtained after the isothermal treatment. NSO means no signal observed.
